# Apoferritin-Encapsulated Jerantinine A for Transferrin Receptor Targeting and Enhanced Selectivity in Breast Cancer Therapy

**DOI:** 10.1021/acsomega.2c00997

**Published:** 2022-06-13

**Authors:** Haneen Abuzaid, Salah Abdelrazig, Lenny Ferreira, Hilary M. Collins, Dong-Hyun Kim, Kuan-Hon Lim, Toh-Seok Kam, Lyudmila Turyanska, Tracey D. Bradshaw

**Affiliations:** † School of Pharmacy, Biodiscovery Institute, The University of Nottingham, University Park, Nottingham NG7 2RD, U.K.; ‡ The University of Nottingham Malaysia, Block B, Malaysia Campus, Jalan Broga, 43500 Semenyih, Selangor, Malaysia; § Department of Chemistry, Faculty of Science, The University of Malaya, 50603 Kuala Lumpur, Malaysia; ∥ Faculty of Engineering, The University of Nottingham, Additive Manufacturing Building, Jubilee Campus, University Park, Nottingham NG7 2RD, U.K.

## Abstract

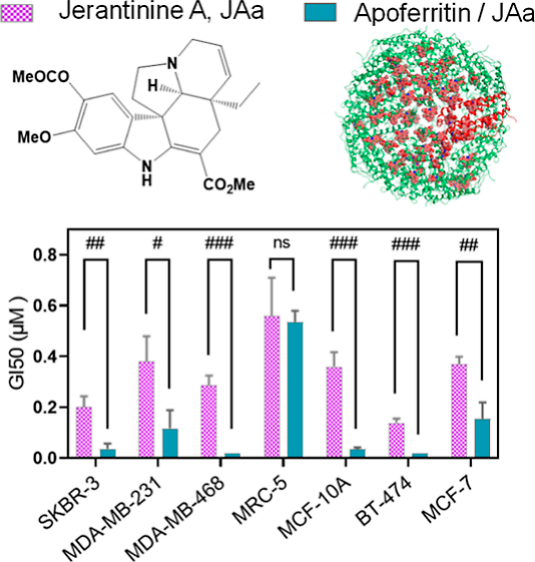

The O-acetyl (or acetate) derivative of the Aspidosperma alkaloid Jerantinine A (JAa) elicits anti-tumor activity against cancer cell lines including mammary carcinoma cell lines irrespective of receptor status (0.14 < GI_50_ < 0.38 μM), targeting microtubule dynamics. By exploiting breast cancer cells’ upregulated transferrin receptor 1 (TfR1) expression and apoferritin (AFt) recognition, we sought to develop an AFt JAa-delivery vehicle to enhance tumor-targeting and reduce systemic toxicity. Optimizing pH-mediated reassembly, ∼120 JAa molecules were entrapped within AFt. Western blot and flow cytometry demonstrate TfR1 expression in cancer cells. Enhanced internalization of 5-carboxyfluorescein-conjugated human AFt in SKBR3 and MDA-MB-231 cancer cells is observed compared to MRC5 fibroblasts. Accordingly, AFt–JAa delivers significantly greater intracellular JAa levels to SKBR3 and MDA-MB-231 cells than naked JAa (0.2 μM) treatment alone. Compared to naked JAa (0.2 μM), AFt–JAa achieves enhanced growth inhibition (2.5–14-fold; <0.02 μM < GI_50_ < 0.15 μM) in breast cancer cells; AFt–JAa treatment results in significantly reduced clonal survival, more profound cell cycle perturbation including G2/M arrest, greater reduction in cell numbers, and increased apoptosis compared to the naked agent (*p* < 0.01). Decreased PLK1 and Mcl-1 expression, together with the appearance of cleaved poly (ADP-ribose)-polymerase, corroborate the augmented potency of AFt–JAa. Hence, we demonstrate that AFt represents a biocompatible vehicle for targeted delivery of JAa, offering potential to minimize toxicity and enhance JAa activity in TfR1-expressing tumors.

## Introduction

Worldwide, breast cancer is the most common malignancy and the leading cause of cancer mortality among women,^1^ with an estimated 684,996 breast cancer deaths globally in 2020. Despite the essential role of chemotherapy in cancer treatment, drug resistance represents a major problem in reducing the efficacy of therapy.^2,3^ Indeed, ∼90% of metastatic cancer patients succumb to chemotherapy failure due to both *de novo* and acquired drug resistance.^4^ Thus, there is an urgent unmet clinical need for the development of new agents and strategies to overcome drug resistance.^5^ Adverse toxicity further thwarts successful chemotherapy: cardiac, hematologic, pulmonary, renal, and many other toxicities result from systemic chemotherapy.^6^ Efforts to enhance efficacy, evade resistance, and reduce toxicity using combination therapies in breast cancer patients have led to better response and survival rates,^7^ but they may also induce greater toxicity compared to single-agent therapies.^8,9^ Targeted delivery of anticancer agents within nanoparticles has been reported to enhance response and reduce toxicity *in vitro* and *in vivo*.^10,11^ Dextran, for example, natural, renewable, biocompatible, and biodegradable, can be applied as a carrier for targeted drug delivery.^12^ Among drug delivery systems, biocompatible and biodegradable protein capsules are of particular interest.^13^ Apoferritin (AFt), with outer and inner diameters of 12 and 8 nm, respectively,^14^ has been investigated for more targeted delivery of anticancer agents, such as navitoclax, temozolomide, doxorubicin, and cisplatin.^15−18^ AFt is recognized by cellular transferrin receptor 1 (TfR1)^19^ that is overexpressed in tumors including breast cancers.^20,21^ TfR1 upregulation is associated with proliferative diseases where iron demand is high—including cancer—in particular areas of tumor hypoxia and is thus an important characteristic of the tumor microenvironment.^22,23^ Hence, targeting TfR1 may be exploited to achieve selective uptake by tumor cells.^20^

Naturally occurring indole alkaloids are an important source of anticancer agents used clinically, exemplified by the *Vinca* alkaloids.^24^ Jerantinines are *Aspidosperma* indole alkaloids isolated from *Tabernaemontana corymbosa* leaves that demonstrated broad-spectrum anticancer activity, perturbing molecular targets pertinent to tumorigenesis.^25^ Crystallography studies demonstrated binding to the colchicine binding site of tubulin, underpinning the profound G2/M cell cycle arrest caused by jerantinines A and B (JA and JB).^26^ Their antiproliferative mechanisms involve inhibiting tubulin polymerization and PLK1 activity while increasing ROS production in treated cancer cells.^27,28^ Significantly, HCT-116 cells, possessing acquired resistance to vincristine, lack cross-resistance to jerantinines. These cells highly express the P glycoprotein (Pgp) multidrug-resistant efflux pump;^27^ therefore, jerantinines are not Pgp substrates and may offer therapy in multidrug-resistant malignancies, for example, as a potential alternative to vincristine in the treatment of Pgp-positive pediatric medulloblastoma.^29^ However, despite the promising activity against diverse human carcinomas, including breast cancer cell lines MCF-7 and MDA-MB-468 (GI_50_ values of <1 μM),^27,28^ jerantinines present potential problems associated with cancer selectivity and putative toxicity, highlighted by the sensitivity of non-cancerous lung (MRC-5) fibroblasts.^27,28^ Hence, cancer selectivity needs to be addressed to advance preclinical development of jerantinines.

Here, we report robust, reproducible entrapment of ∼150 molecules of jeratinine A acetate (JAa) within AFt, optimizing the pH-mediated disassembly/reassembly route and overcoming a major drawback associated with this method—formulation yield.^30^ Thorough characterization of the nanoformulation is described. We demonstrate significantly enhanced cancer selectivity and antitumor activity in breast cancer cell lines of distinct phenotypes following AFt-encapsulation of JAa, confirming that cell cycle blockade and apoptosis-induction by JAa are potentiated following AFt-encapsulation. Demonstrating (i) TfR-1 expression in our breast cancer cell line panel irrespective of ER, PR, and HER2 expression; (ii) AFt-uptake by breast cancer cell lines; and (iii) greater intracellular accumulation of its cargo, JAa, in cancer cells compared to non-transformed MRC5 fibroblasts, we provide evidence that enhanced activity is a consequence of TfR-1-mediated internalization of AFt (and its encapsulated cargo). Thus, we propose an AFt-encapsulation JAa formulation for enhanced targeting of JAa to cancers expressing TfR-1 and demonstrate its potential for wide-spectrum activity against multiple breast cancer phenotypes.

## Materials and Methods

### Preparation of AFt

AFt was prepared from horse spleen ferritin (Ft) (Sigma-Aldrich) as described originally by Granick and Michaelis in 1943 with some modifications.^14^ Briefly, diluted horse spleen Ft in sodium acetate buffer (NaOAc, 0.1 M, pH 5.5) was dialyzed against 2 L of the same buffer for 3 h. Mercaptoacetic acid (3 mL) was used as a reducing agent in the outer buffer system. The dialysis process was repeated 5 times until a colorless protein was obtained. Dialysis was carried out under constant N2 purge in the fume hood. Ultrapure water (18.2 MΩ cm) was used in all experiments. All chemical reagents were used as received without further purification.

### AFt-Encapsulation of JAa: Reassembly Method

JAa was synthesized from sustainably sourced tabersonine adopting a straightforward, robust procedure previously described.^26^ The previously prepared AFt stock was diluted using ultrapure water to a concentration of 2.5 mg/mL. AFt was disassembled into subunits by adding HCl (1 M) to achieve pH 2. The solution was kept on ice and stirred for 30 min. Subsequently, JAa was added at a ratio of 150 molecules to each AFt molecule. The addition of JAa was performed slowly alongside NaOAc buffer (0.1 M, pH 4) to reassemble the protein to its original cage. After adjusting the solution with NaOAc to pH 4, it was kept under stirring conditions at 4 °C for another 60 min. The protein then was fully reassembled by adding tris-acetate buffer (0.1 M, pH 7.4) and kept at 4 °C under stirring overnight. The solution was centrifuged at 3000 rpm for 15 min at 4 °C using Amicon Ultra Centrifugal Filters with a molecular weight cutoff (MWCO) of 100 kDa to remove aggregated protein and un-encapsulated drug molecules. The resulting AFt–JAa solution was dialyzed overnight against tris-acetate buffer (25 mM, pH 7.4) and stored at 4 °C for subsequent characterization.

### Protein Recovery and Encapsulation Efficiency

The Bradford assay was used to measure protein concentration before and after the purification step. This was done to assess protein recovery (%) upon the encapsulation of JAa through the reassembly route. The absorbance of JAa encapsulated in AFt was measured using a Varian Cary50, UV–vis spectrophotometer. First, the wavelength at maximum absorbance (λ_max_) was determined by scanning JAa over a wavelength range (200–800 nm). The concentration of JAa was then determined by absorbance at λ_max_ = 330 nm, followed by using a calibration curve (*R*^2^ > 0.99) obtained from known serial dilutions of JAa. Encapsulation efficiency (EE %) was defined as the ratio between the encapsulated and original amount of JAa added to the formulation. Drug loading was calculated as DL (%) = (number of drugs encapsulated × drug MW)/(number of drugs encapsulated × drug MW) + AFt MW) × 100%.

### Native and SDS–PAGE

Native polyacrylamide gel electrophoresis (PAGE) 4–16% bis-Tris gel electrophoresis was used to detect any conformational fluctuations for AFt resulting from the encapsulation of JAa. Cathode (50 mM Tricine, 15 mM BisTris/HCl, pH 7.0) and anode (50 mM BisTris/HCl, pH 7.0) buffers were used to run the gel at 175 mV for 2 h. To preserve the protein in a non-denaturing condition, preparation of samples, sample loading, and gel electrophoresis were performed at 4 °C. The protein bands were visualized by both Coomassie Brilliant Blue staining G250 and under UV light. For sodium dodecyl sulfate (SDS)–PAGE, protein samples were denatured using β-mercaptoethanol, heated for 5 min at 95 °C. Samples were loaded into 15% SDS–PAGE gel chambers, and electrophoresis was performed using Tris–HCl buffer for 1 h at 150 V.

### DLS Measurements

Particle size and surface charge were measured by dynamic light scattering (DLS) techniques using a ZetaSizer Nano ZS, (Malvern Instruments). The AFt–JAa formulation was diluted in deionized water, measured, and compared with AFt alone under the same dilution conditions. The full particle size distribution is reported as the number average.

### Stability under Storage Conditions

The stability of the AFt–JAa formulation at 4 °C was assessed over 10 months. Changes in the particle size and surface charge were evaluated using DLS measurements. The stability of the encapsulated JAa was also evaluated using a UV–vis spectrophotometer to detect changes in the JAa concentration over 5 weeks.

### Stability under Treatment Conditions

The stability of JAa and AFt–JAa at 37 °C was assessed over 72 h. Changes in the concentration of JAa and its hydrolyzed form JA were monitored using LC–HRMS as described later. JAa or AFt–JAa (0.4 μM) was incubated in different media at 37 °C (*n* = 4). Samples were collected at different time intervals (0, 24, 48, and 72 h) and stored at −80 °C prior to LC–HRMS analysis.

### Cell Culture

Based on Perou and Sorlie characterization, one breast cell line was chosen for each major subtype;^31,32^ MCF-7 for luminal A (strongly ER+ and PR+), BT-474 for luminal B (lower ER, PR expression, and HER2+), MDA-MB-468 for basal-like, TNBC (EGFR+), SKBR-3 for HER2 overexpression (ER–, PR–, and HER2+), MDA-MB-231 for claudin-low, TNBC (EGFR+), and MCF-10A for normal-like phenotype. Non-transformed fetal lung MRC-5 fibroblasts were used for comparison. All cell lines were purchased from the American Type Culture Collection (ATCC), subcultured according to ATCC protocols in their appropriate media (Supporting Information, SI3) and checked regularly for mycoplasma infection using a qPCR Kit (Microsart).

### Western Blot Analysis

Cell lysates containing 40 or 50 μg of protein were loaded into 10% SDS–PAGE and run at 175 mV for 2 h. Bradford assays were performed to quantify the protein content.^33^ Proteins were then transferred to a nitrocellulose membrane using the Trans-Blot Turbo Transfer System (Bio-Rad; 2.5 A; 25 V for 45 min) and blocked in 5% low-fat milk at room temperature for 1 h. Subsequently, membranes were incubated in 1° antibody (Ab) overnight at 4 °C, followed by the 2° Ab for 1 h at room temperature. 1° Abs, anti-GAPDH (housekeeping protein), anti-TfR1/2, anti-HER-2, whole poly (ADP-ribose)-polymerase (PARP), cleaved PARP, myeloid cell leukemia 1 (Mcl-1), Bcl-2, and polo-like kinase-1 (PLK-1), purchased from Cell Signaling Technology, and anti-SCARA5, purchased from R&D Systems, were used at a 1:1000 dilution. Anti-rabbit/mouse IGg 2° Ab (Dako) was used at a 1:4000 dilution. All Abs were diluted in 5% low-fat milk. After washing with tris buffered saline-Tween 20, the ECL reagent (GE Healthcare) was used to enable detection of the blots using a Li-COR imaging system. Densitometry analysis of blots was assessed using FIJI software.

### MTT Assay

The MTT assay was used to evaluate the growth and viability of all cell lines used upon treatment with AFt, JAa, and AFt–JAa. Briefly, cells were seeded at a density of 3–5 × 10^3^ per well into 96-well microtiter plates and allowed to attach overnight. All cell lines were treated for 48 h except for BT-474, which was treated for 72 h, and estimated GI50 values were calculated using the GI50 equation (Supporting Information, SI3). The selectivity index (SI) was estimated according to the following calculation: SI = GI50 MRC-5/GI50 breast cancer cell line to indicate the selectivity of JAa and AFt-encapsulated JAa for breast cancer cells over non-cancerous lung (MRC-5) fibroblasts.^34^

### *In Vitro* Cell Viability

Viable cell counts were performed in SKBR-3 and MDA-MB-231 cells to validate growth inhibition observed in MTT assays. Cells, at a density of 2 × 10^4^, were seeded in six-well plates and incubated overnight before treatment. Cells were then exposed to JAa, AFt–JAaAFt–JAa (0.2 μM JAa), AFt (0.0017 μM), or appropriate media alone for 48 h. Cells were then harvested, stained with trypan blue, and counted using a hemocytometer.

### Clonogenic Assay

In six-well plates, 700 SKBR-3 and BT-474 cells were seeded per well and (after overnight incubation) treated for 48 h with JAa, AFt–JAa (0.2 μM and 0.4 μM JAa), AFt (0.0033 μM), or medium alone. Cells were then washed with phosphate-buffered saline (PBS), replaced with fresh medium, and incubated at 37 °C. Assays were terminated when colonies of ≥50 cells were observed in control wells. Colonies were fixed (100% methanol), stained (0.05% methylene blue), and counted and survival fractions (SF %) calculated.

### Flow Cytometry

A Beckman Coulter FC500 flow cytometer was used to run samples, and fluorescence signals were detected using channel FL2 (575 nm/40 detector) for TfR1 expression, and cell cycle assessment, FL1(525 nm/40 detector) for carboxyfluorescein-conjugated human H-AFt, while both FL2 and FL3 (620 nm/20 detector) channels were used for the Annexin-V/propidium iodide (PI) apoptosis assay. For each sample, 20,000 single cells were gated for analysis.

### Assessment of TfR1 Expression

To determine TfR1 expression levels, cells were labeled with human phycoerythrin (PE)-conjugated anti-CD71 (Invitrogen) 1° Ab. Cells were seeded at a density of 1 × 10^5^ in six-well plates and allowed to attach overnight. Cells were harvested after trypsinization, washed with PBS, and blocked with 1% fetal bovine serum (FBS)/PBS for 30 min at room temperature. After washing again with PBS, cells were pelleted and stained with anti-CD71 (2% in 1% FBS/PBS for 45 min at 4 °C). Cells were fixed with 3.7% formaldehyde/PBS for 5 min at room temperature before flow cytometry analysis.

### Cell Cycle Analysis

SKBR-3 and MDA-MB-231 cells at a density of 1 × 10^5^ were seeded in 6-well plates. After overnight incubation, cells were treated with JAa, AFt–JAa (0.2 μM and 0.4 μM JAa), AFt (0.0033 μM), or appropriate media alone for 48 h. Cells were then harvested and washed with PBS by centrifugation (1200 rpm, 5 min, 4 °C). The pellets were re-suspended in 0.5 mL of ice-cold hypotonic fluorochrome solution (50 μg/mL PI, 0.1 mg/mL ribonuclease A, 0.1% v/v Triton X-100, and 0.1% w/v sodium citrate). Cells were then incubated overnight at 4 °C before flow cytometry analysis.

### Annexin V-FITC and PI Apoptosis Assays

SKBR-3 and MDA-MB-231 cells were seeded, treated, harvested, and pelleted as per cell cycle analysis. Using the Annexin V-FITC apoptosis detection kit (BD Pharmingen), cells were incubated in the dark with 5% annexin V-FITC [100 μL, 15 min, retention time (RT)] followed by reincubation with 2.5% PI solution (400 μL, 10 min, RT) and analyzed by flow cytometry within 1 h of the preparation.

### Cellular Uptake of Human-AFt

To correlate the cellular uptake of AFt-encapsulated agents to TfR1 expression in SKBR-3, MDA-MB-231, and MRC-5 cells, cells at a density of 5 × 10^4^ were exposed to 5-carboxyfluorescein-conjugated H-AFt at a concentration of 40 nM and incubated at 37 °C for the desired time (1, 2.5, 4, and 24 h). The cells were then harvested after trypsinization and washed with PBS before pelleting and fixing with 3.7% formaldehyde/PBS for 5 min. Cells were pelleted again and suspended in PBS before analysis by flow cytometry.

### Cellular Uptake of Free and Encapsulated JAa

To correlate the cellular uptake of the AFt-encapsulated agent to the detected enhanced activity, SKBR-3 and MDA-MB-231 cells at a density of 2 × 10^6^ were exposed to JAa, AFt–JAa (0.4 μM JAa), AFt (0.0033 μM), or appropriate media alone for 72 h (*n* = 7). Following treatment, growth medium/floating cells were collected for extracellular samples and centrifuged at 13,300 rpm for 10 min at 4 °C. Supernatants were mixed with pre-cooled methanol at a 1:3 ratio and incubated for 20 min (−20 °C) to precipitate proteins. Samples were centrifuged at 13,300 rpm for 10 min at 4 °C before analysis of supernatants with LC–HRMS. For intracellular samples, the treated cells were quenched with pre-cooled methanol (1 mL, ≤ −48 °C), scraped, and transferred into pre-cooled tubes (4 °C). The cell extracts were vigorously vortexed for 1 h and then centrifuged (HERAEUS-Fresco 17 centrifuge; Thermo Election Corporation) at 13,300 rpm for 10 min at 4 °C. The supernatants were transferred into pre-cooled tubes and completely dried under vacuum using a Jouan RC10.22 vacuum concentrator (Thermo Scientific). The samples were then reconstituted in methanol (70 μL) and analyzed with LC–HRMS.

### Release Kinetic Studies

JAa release from the AFt–JAa formulation was conducted using an A-Lyzer MINI Dialysis Device, MWCO 10 kDa (Thermo Scientific). The formulation (*n* = 3) was placed into the device slide and kept at 37 °C in either NaOAc buffer (pH 5.3) or PBS (pH 7.4). Samples were collected at different time intervals (0, 1, 3, 6, and 24 h), and the concentration of JAa was quantified using LC–HRMS and a calibration curve of JAa authentic standard (0.195–50 μM).

### Liquid Chromatography–High-Resolution Mass Spectrometry

LC–HRMS quantification was performed using a Dionex U3000 UHPLC system (Thermo Fisher Scientific, Hemel Hempstead, UK) on a ZIC-pHILIC column (4.6 × 150 mm, 5 μm particle size, Merck SeQuant, Gillingham, UK), as previously described with a small modification.^35^ Briefly, the column was maintained at 45 °C at a flow rate of 300 μL/min. Mobile phases used were (A) 20 mM ammonium carbonate and (B) acetonitrile. The gradient started with 20% (A) and increased to 95% (A) over 8 min, then the composition was returned to 20% (A) in 2 min at 400 μL/min, and then the column was left to re-equilibrate under the initial gradient conditions for 5 min (15 min total time). The injection volume was 5 μL, and samples were maintained at 4 °C during the analysis. An orbital trap mass spectrometer (QExactive-Orbitrap, Thermo Fisher Scientific) was used in simultaneous ESI^+^ and ESI^–^ modes for LC–HRMS quantification. The operational parameters were spray voltage 4.5 kV (ESI^+^), 3.5 (ESI^–^), capillary voltage 20 V (ESI^+^), −15 V (ESI^–^), sheath, auxiliary, and sweep gas flow rate were 40, 5, and 1 (arbitrary unit), respectively, for both modes. Capillary and heater temperatures were maintained at 275 and 150 °C, respectively. Data were acquired in full scan mode with a resolution of 70,000 from *m*/*z* 70 to 1050. Data-dependent tandem MS (ddMS/MS) scans were also obtained on the samples and the authentic standard at a resolution of 17,500 and a stepped normalized collision energy of 20, 30, and 40 for the structural confirmation of JAa using the RT and fragmentation pattern based on MS/MS.

### Statistical Analysis

Results were evaluated using Graph Pad Prism v9.1.0. Representative figures are shown as median ± standard deviation (SD) for flow cytometry, and as mean ± SD for all other experiments. One- and two-way ANOVA statistical analyses and Sidak’s multiple comparisons test were used to assess significant differences and defined as * (*p* < 0.05), ** (*p* < 0.01), *** (*p* < 0.001), and **** (*p* < 0.0001); significant. Differences between encapsulated and naked JAa are expressed as # (*p* < 0.05), ## (*p* < 0.01), ### (*p* < 0.001), and #### (*p* < 0.0001). All experiments were repeated ≥3 times with internal replicates of *n* ≥ 3.

## Results and Discussion

**Figure 1:**
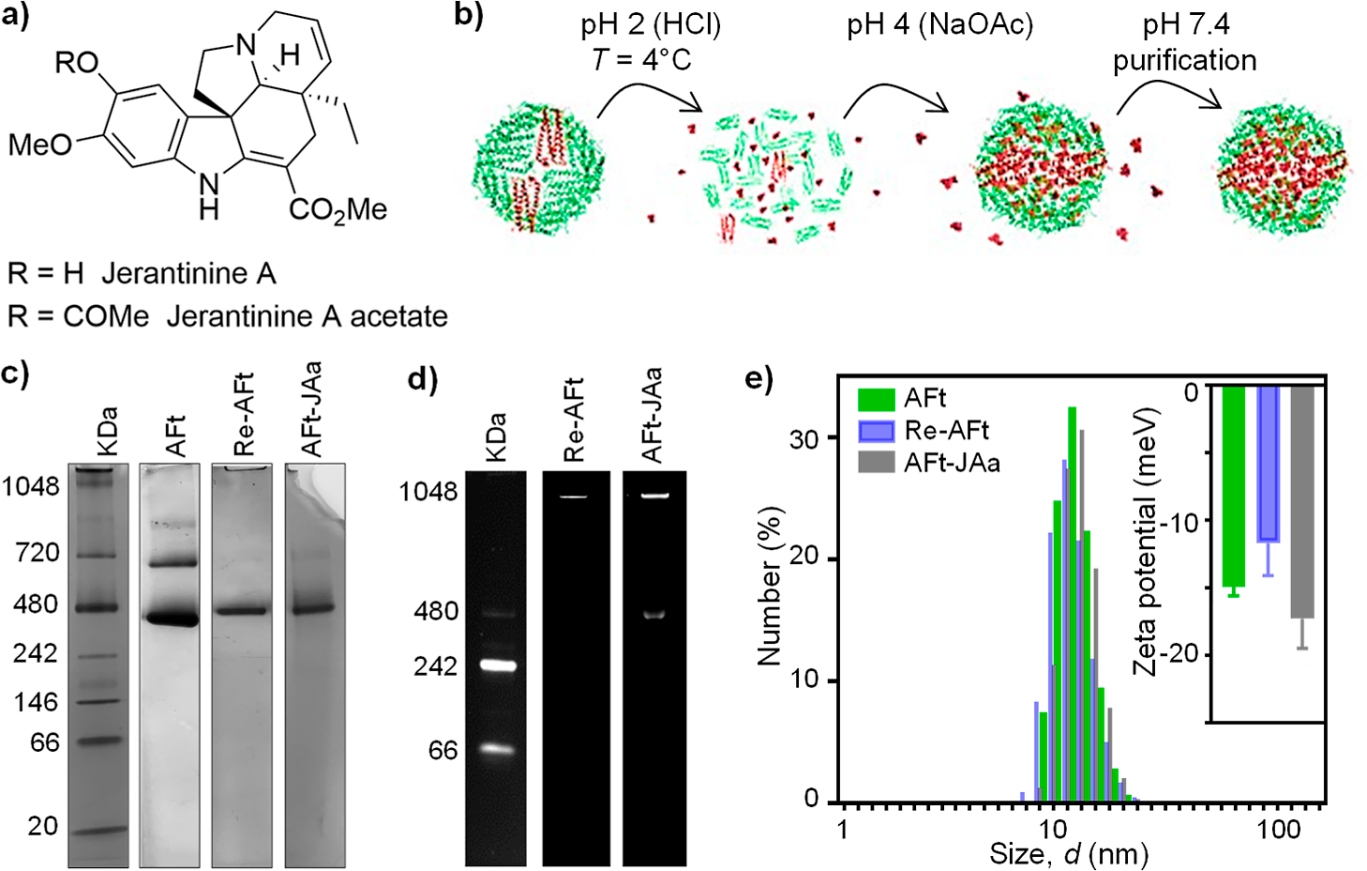
(a) Chemical structure of JAa and JAa acetate. (b) Illustration of AFt-encapsulation of JAa via reassembly. (c,d) Characterization of AFt integrity: AFt stock, reassembled AFt without a drug (Re-AFt), and AFt-encapsulated JAa (AFt–JAa) (c) using (4–16%) polyacrylamide blue native gel electrophoresis and (d) under UV light. (e) DLS analyses of the hydrodynamic diameter shown as the number (%) and zeta potential of AFt, Re-Aft, and AFt–JAa.

To minimize potential cytotoxicity and enhance cancer cell selectivity, the acetate derivative of aspidosperma indole alkaloid JA isolated from *T. corymbosa* was encapsulated inside the core of AFt (Figure 1). The well-understood pH-mediated self-assembly method reported previously^36^ for encapsulation of different agents was adapted and optimized.^30^ Briefly, horse spleen AFt (2.5 mg/mL) was disassembled into its subunits using HCl to reduce the pH of the solution (2.0–2.5). The protein cage was reassembled in the presence of JAa using a two-step pH change: initially from pH 2 to pH 4 followed by an increase to pH 7.4. The AFt–JAa formulation produced with an AFt/JAa ratio of 1:150 achieved encapsulation of 120 molecules per cage, as assessed by UV–vis spectroscopy (Supporting Information, SI1), corresponding to EE = 80 ± 7% (Table 1). The Bradford assay was used to quantify protein recovery and 77 ± 5 and ∼70% were achieved for AFt and AFt–JAa, respectively, which are higher than that reported previously (64%).^30^ In this method, a gradual pH increase is used to yield higher protein recovery compared to one-step pH change disassembly methods with HCl or glycine acetate buffer.

**Table 1:** Summary of Characterization Data for AFt Stock, AFt after Reassembly and AFt–JAa Produced with an AFt/JAa Ratio of 1:150^a^

	AFt stock	AFt	AFt–JAa
size by number (nm)	12.13 ± 0.4	12.5 ± 0.5	13.5±0.3
zeta potential (mV)	–14.9 ± 0.7	–11.7 ± 2.4	–17.3±2.2
protein recovery (%)		77 ± 5	69 ± 7
EE (%)			80 ± 7
number of JAa molecules per AFt			120 ± 10
DL			9.5%

a Results represent mean ± SD from ≥3 independent trials.

Reassembly, with integrity, of AFt empty capsules as well as following successful entrapment of JAa is demonstrated by native PAGE (Figure 1c). An intense band was observed corresponding to molecular weight, MW ∼480 kDa expected for AFt,^37^ confirming successful reassembly of the cage. Under UV light illumination, the fluorescence of this band indicates the presence of JAa, confirming successful encapsulation of the agent inside the AFt cage (Figure 1d). DLS studies revealed the hydrodynamic size *d* = 13.5±0.3 nm and zeta potential ζ = −17.3±2.2 mV for AFt–JAa comparable to that of AFt before the encapsulation process, *d*_AFt_ = 12.13 ± 0.4 nm and ζ = −14.9 ± 0.7 mV (Table 1 and Figure 1e). Analysis of morphology and composition of AFt–JAa using a combination of native PAGE, DLS, and UV–vis spectroscopy provides convincing, conclusive evidence of successful encapsulation of JAa within the AFt core.

Release studies were performed under physiologically relevant conditions *T* = 37 °C, normal pH 7.4, and pH 5.3 associated with the lysosome environment. Samples were collected at different time points over a 24 h study and were analyzed by LC–HRMS. The results showed that within the first 6 h, the release rate was ∼23% slower at pH 5.3 compared to pH 7.4. After 24 h, ∼2% and ∼4% JAa remained within the AFt core at pH 7.4 and pH 5.3, respectively (Supporting Information, SI4; Figure S3). The slower release at pH 5.3 can be explained by the p*K*_a_ of JAa of ∼7.5, which is >2 units higher than the pH; hence, according to the Henderson–Hasselbalch equation,^38^ over 99% of JAa molecules will be protonated at this pH. The positively charged JAa molecules will be attracted to the negatively charged AFt interior, leading to slower release compared to that at physiological pH 7.4. AFt–JAa formulations remain stable when stored at *T* = 4 °C ≤ 10 months as confirmed by DLS and UV–vis studies. However, JAa started to degrade after 4 weeks; therefore, fresh formulations were prepared every month for *in vitro* assessments (see Supporting Information, SI5).

**Figure 2:**
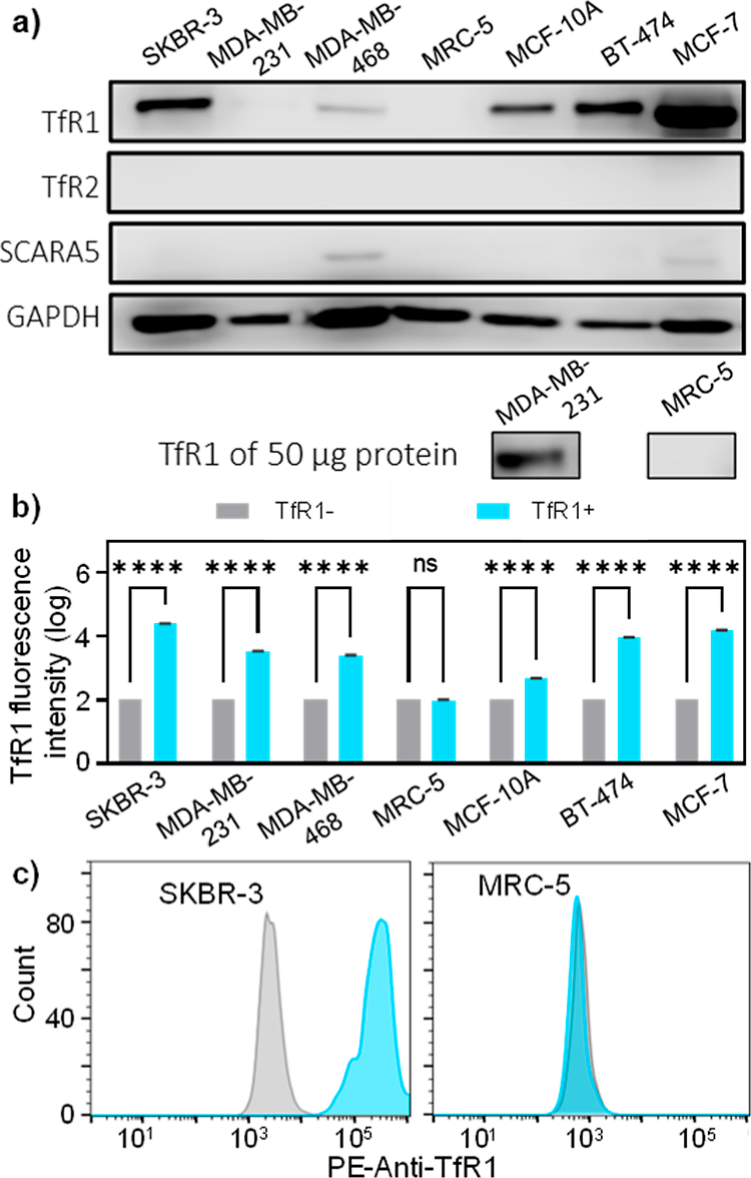
(a) Immunoblotting for whole (intracellular and extracellular) TfR1 in cell lysates containing 40 or 50 μg of protein confirms its strong expression in SKBR-3, MCF-10A, BT-474, and MCF-7, weak expression in MDA-MB-231 and MDA-MB-468, and non-detectable expression of TfR1 in MRC-5 lysates. Immunoblotting also shows weak expression of the SCARA-5 receptor in MDA-MB-468 and MCF-7 lysates only. (b) Expression of TfR1 on the surface of cell lines showing a significant shift in TfR1 after binding of PE-Anti-TfR1 in all tested breast cell lines (SKBR-3, MDA-MB-231, MDA-MB-468, MCF-10A, BT-474, and MCF-7), MRC-5 shows no significant shift. (c) Representative histograms of TfR1+SKBR-3 and TfR1-MRC-5 cells. Values are reported as median ± SD (*n* = 3). ****p < 0.0001.

To validate TfR1-targeting using AFt nanoparticles, TfR1 expression in all used cell lines was first investigated (Figure 2). Western blot analyses detected diverse expressions of TfR1 in breast cell line lysates containing 40 μg of protein (Figure 2a). These included SKBR-3, MDA-MB-468, BT-474, and MCF-7 cancer, non-transformed immortalized epithelial MCF-10A cells, as well as in MDA-MB-231 lysate containing 50 μg, whereas in MRC-5 fibroblast lysates of 40 and 50 μg of protein, expression of TfR1 was below detectable levels. Immunoblotting also showed weak expressions of SCARA-5 in MDA-MB-468 and MCF-7; no expression of TfR2 was detected in any of the cell lines.

The levels of TfR1 were also measured by flow cytometry after labeling the cells with PE-conjugated anti-TfR1 (Figure 2b,c). For MRC-5 fibroblasts, the shift in the TfR1 signal was indistinguishable from the control un-labeled cells; hence, MRC5 cells were thus termed TfR1–. In contrast, significantly higher shifts in TfR1 were detected in all breast cell lines (*p* < 0.0001). SCARA-5 and TfR2 receptors contribute to the intracellular uptake of ferritin through the recognition of the L-chain and H-chain, respectively.^39,40^ Weak or undetectable expression levels of both receptors suggest that the uptake of AFt is primarily via TfR1. We note that TfR1 overexpression, confirmed in breast cancer cells by both immunohistochemical semi-quantitative analysis and flow cytometric analysis, provides an exceptional opportunity for active targeting by AFt.^19,20,41^ TfR-1-MRC5 cells provide an appropriate negative control for our studies; however, it should be cautioned that upregulated TfR1 expression may also be associated with benign proliferative pathologies such as mammary gland fibrocystic disease as exemplified by non-tumorigenic MCF-10A cells.^42^

**Figure 3:**
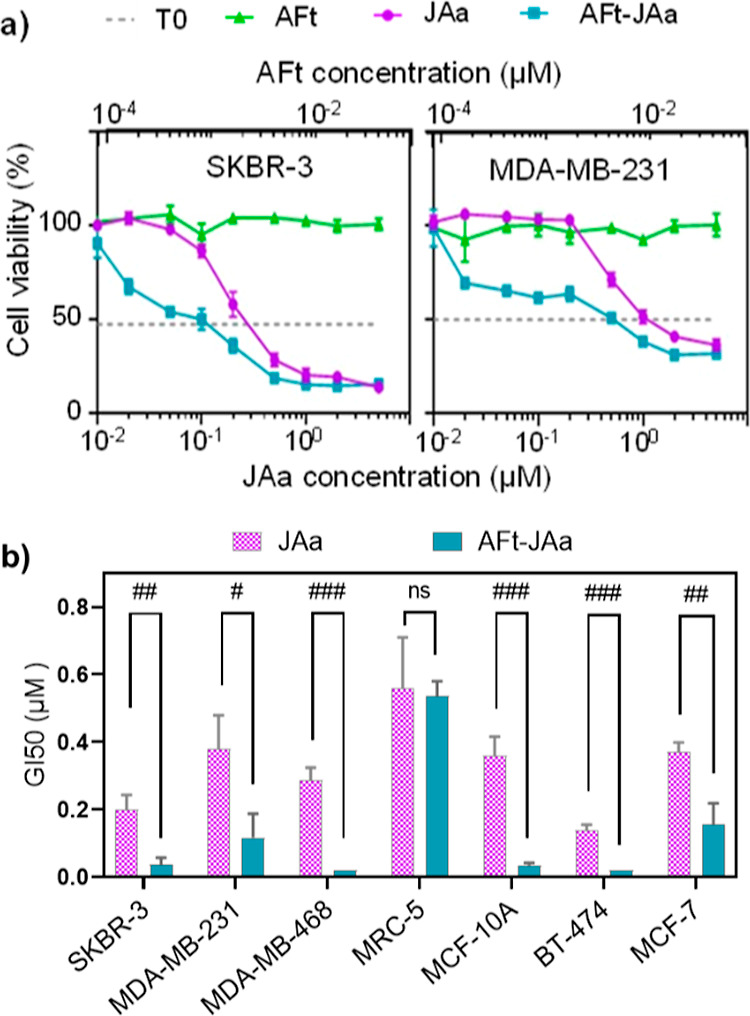
(a) Representative MTT graphs from a single experiment (*n* = 4 internal replicates) displaying the growth inhibitory properties of AFt-encapsulated JAa (AFt–JAa), JAa, and AFt in SKBR-3, MDA-231 cell lines. TO = absorbance (550 nm) at the time of test agent addition representing viability/viable cell numbers prior to treatment. (b) Comparative GI_50_ profiles in all studied cell lines after treatment with JAa and AFt–JAa. Statistical analyses revealed significant reduction in GI_50_ values in all cell lines except MRC-5. Values are reported as mean ± SD (*n* ≥ 3 independent trials). #*p* < 0.05, ##*p* < 0.01, and ###*p* < 0.001.

The effects of AFt encapsulation on JAa activity were evaluated *in vitro* using MTT, cell count, clonogenic assays, cell cycle, and apoptosis analyses. For cell viability, MTT assays were first adopted. SKBR-3, MDA-MB-231, MDA-MB-468, MCF-7, MCF-10A, and MRC-5 cells were treated with JAa or AFt–Jaa, with JAa concentrations in the range 0.02–5 μM JAa. All cell lines with the exception of BT-474 were exposed to test agents for 48 h. BT-474 cells, because of their slow doubling time (>48 h), were exposed to test agents for 72 h. Bearing in mind the fact that Jaa is a microtubule destabilizing agent, an exposure period of at least one doubling time is necessary to capture cells traversing mitoses. Concentration-dependent growth inhibition was observed with naked Jaa, demonstrating activity against all breast cancer cell lines (Figures 3a and Supporting Information, S5). Selectivity for cancerous breast cell lines over non-cancerous MCF-10A cells was detected only at high JAa concentrations of 5 μM, where growth inhibition of 98 and ∼86% was observed in breast cancer MDA-MB-468 and SKBR-3 cells, respectively, compared to ∼52% in non-cancerous MCF-10A cells (Supporting Information, Figure S6b,c). These data indicate some cancer selectivity of JAa.^43^ However, JAa non-selectively inhibited the growth of MRC-5 normal fibroblasts at the same concentration of 5 μM by ∼91% (Supporting Information, Figure S6d), as was previously reported for JB and JA,^27,28^ presenting challenges for its applications.

Following treatment with AFt–JAa, growth inhibition in all cell lines was evident even at the lowest treatment concentration of 0.02 μM JAa (Figures 3a and Supporting Information, S5). Significant reductions in GI_50_ values (*p* < 0.05) were observed following treatment with AFt–JAa compared to the naked agent (Table 2 and Figure 3b). For example, >7-fold and >14-fold reductions in GI_50_ values were recorded for BT-474 and MDA-468 cells, respectively, with GI_50_ < 0.02 μM for Aft–Jaa in both cell lines. At higher concentrations (≥2 μM Jaa), comparable growth inhibition was observed following treatment with both naked and AFt-encapsulated JAa (Figures 3a and Supporting Information, S5).

**Table 2:** Summary of GI_50_ and SI Values of JAa and AFt–JAa in All Studied Cell Lines

cell lines	SKBR-3	MDA-231	MDA-468	MRC-5	MCF-10A	BT-474	MCF-7
GI_50_ (μM) ± SD for *n* = 3							
JAa	0.19 ± 0.04	0.38 ± 0.10	0.28 ± 0.04	0.56 ± 0.15	0.36 ± 0.05	0.14 ± 0.02	0.37 ± 0.03
AFt–JAa	0.04 ± 0.01	0.12 ± 0.07	<0.02	0.53±0.04	0.03 ± 0.01	<0.02	0.15 ± 0.06
fold enhanced activity	4.75	3.17	>14	1.06	12	>7	2.47
SI							
JAa	2.9	1.5	2.0		1.6	4.0	1.5
AFt–JAa	13.3	4.4	>26.5		17.7	>26.5	3.5
TfR1	250	33	24		4	92	158

We propose that at lower concentrations, AFt potentiates intracellular delivery of JAa, whereas at high JAa concentrations, the tubulin-binding site of JAa may be saturated and no further selectivity can be achieved. For non-tumorigenic TfR1-MRC-5 fibroblasts, the mean GI_50_ value for AFt–JAa (0.53 ± 0.04 μM) was comparable to that for JAa alone. Importantly, exposure of cells to AFt alone did not affect the growth or viability in any cell line. SIs were calculated for JAa and AFt–JAa (Table 2). After 48 h of exposure, good SI values for AFt–JAa in all TfR1+ cell lines were detected (SI values ∼3.5 to ∼26.5). Of note, SI values calculated for JAa were ≤4, confirming the potential of AFt-encapsulation to target TfR1-overexpressing cell lines. To corroborate data from MTT assays, *in vitro* viable cell count assays were conducted in two TfR1+ breast carcinoma cell lines (SKBR-3 and MDA-MB-231) following treatment with naked- or AFt–JAa (0.2 μM JAa). Significant reduction of viable cell numbers was observed in SKBR-3 and MDA-MB-231 cells following exposure to AFt–JAa compared to naked JAa, *p* < 0.001 and *p* < 0.05, respectively. Exposure of cells to AFt alone (0.0017 μM) did not cause any detrimental effect on viable cell numbers (Supporting Information, Figure S8).

**Figure 4:**
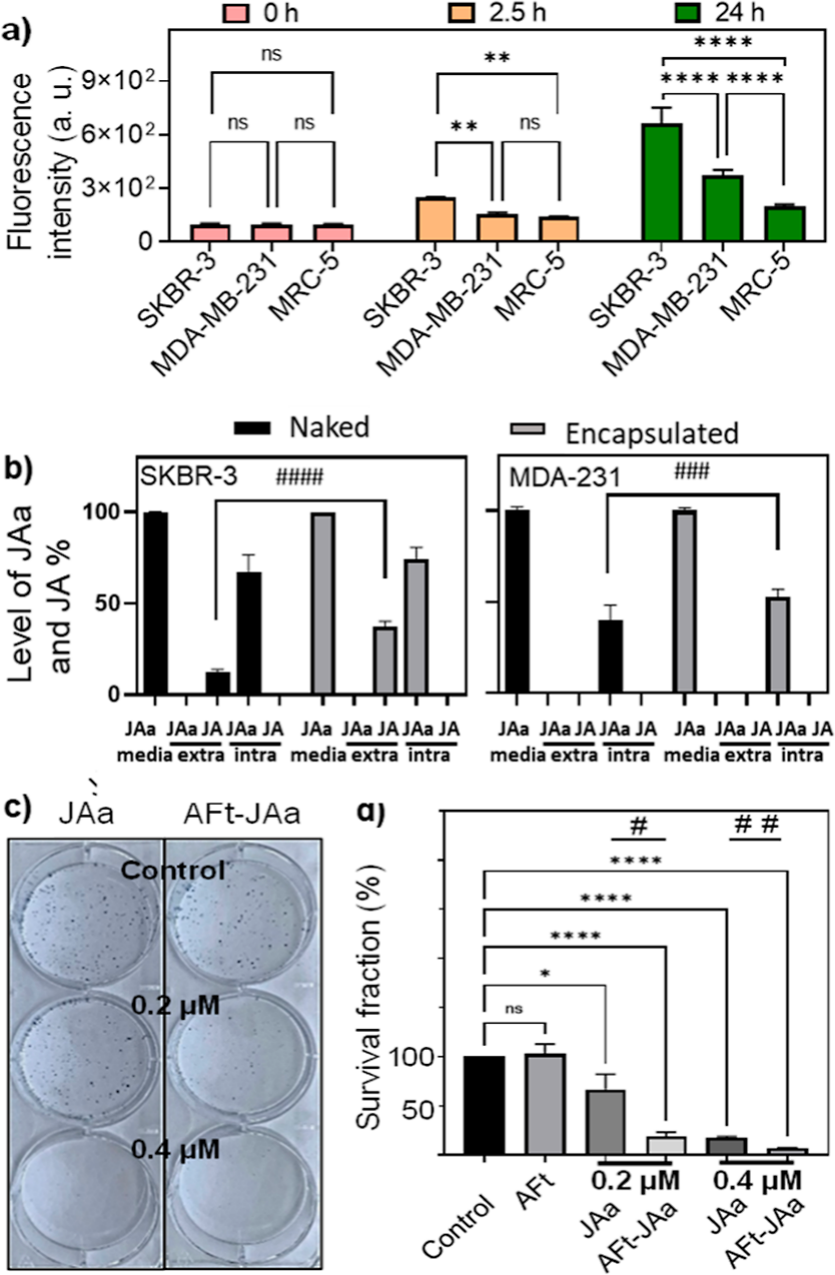
(a) Histograms of cellular uptake in SKBR-3, MDA-231, and MCR-5 cells of 5-carboxyfluorescein-conjugated human-AFt (40 nM) following up to 24 h exposure. Data are reported as mean ± SD (*n* = 3) repeated 3 times. Significant differences from non-cancerous MRC-5 cells are expressed as *p < 0.05, **p < 0.01, and ****p < 0.0001. (b) Intra- and extra-cellular levels of JAa and its hydrolyzed form following 72 h 0.4 μM exposure to JAa and AFt–JAa. Data are represented as mean ± SD (*n* = 7). Significant differences from naked JAa are expressed as ###*p* < 0.001. ####*p* < 0.0001. (c) Representative SKBR3 colonies demonstrating the effect of naked or AFt-encapsulated JAa (0.2 and 0.4 μM JAa), AFt (0.0033 μM), or medium alone on SKBR-3 clonal survival following 48 h of exposure. (d) Clonogenic SFs reported as mean ± SD (*n* = 3), repeated 3 times. Significant differences are expressed as *p < 0.05 and ****p < 0.0001. Significant differences from naked JAa are expressed as #*p* < 0.05 and ##*p* < 0.01.

To examine further the enhanced activity of AFt–JAa and explore the correlation with TfR1 expression, cellular AFt-uptake was assessed by flow cytometry. The fluorescence of 5-carboxyfluorescein conjugated to H-AFt after 1, 2.5, 4, and 24 h of treatment of cells with a 40 nM agent (equivalent to the highest concentration of AFt used in the MTT assay) was measured in SKBR-3, MDA-MB-231 (TfR1+), and MRC-5 fibroblasts (TfR1-). The fluorescence signal in all studied cells increased with increasing exposure time, confirming accumulation of H-AFt (Figures 4a and Supporting Information, S11). However, after 2.5 h of treatment, 1.5-fold enhanced H-AFt uptake was observed in SKBR-3 (TfR1+); in MRC-5 cells, only 0.4-fold increase was observed compared to their untreated controls. Following 24 h of treatment, 5.6-fold and 2.7-fold increased uptake was observed in TfR1+ SKBR-3 and MDA-MB-231 cells, respectively, compared to an ∼2-fold increase in MRC-5 fibroblasts. Both TfR1+ breast cancer cell lines demonstrated greater H-AFt uptake compared to TfR1- MRC-5 cells, supporting a role for TfR1 receptor-mediated cellular uptake of AFt and growth inhibition observed following exposure of these cancer cells to an AFt-encapsulated agent.

The results are consistent with ∼250- and 33-times higher expression of TfR1 (Figure 2) and SI values of ∼13.5 and ∼4.5 (Table 2) in SKBR3 and MDA-MB-231 cells, respectively, compared to non-cancerous MRC-5 fibroblasts. We also detect that the TfR1-recognition can be abolished through mutagenesis of AFt at the TfR1 binding recognition site (unpublished results). Following 72 h of exposure of cells to 0.4 μM free and encapsulated JAa, LC–HRMS analyses of JAa and its hydrolyzed form JA revealed greater intracellular JAa levels, by 7 and 13% in SKBR-3 and MDA-MB-231 cells, respectively, following treatment with AFt-encapsulated JAa (Figure 4b). Moreover, at 72 h, 25% higher levels of extracellular JA were detected in SKBR-3 culture exposed to AFt–JAa, reflecting greater JAa stability afforded by AFt-encapsulation (Supporting Information, Figure S4d).

**Figure 5:**
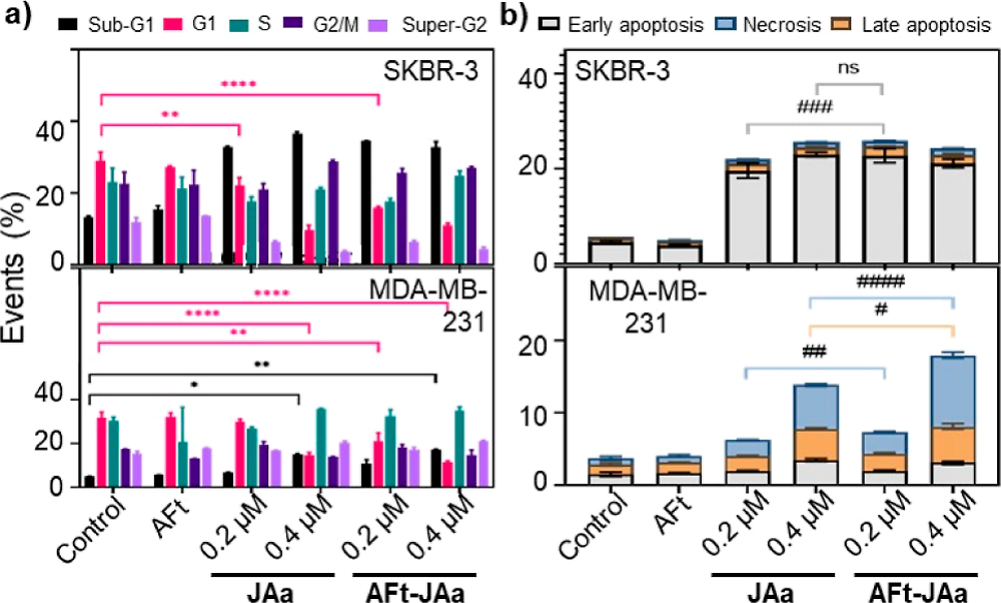
Effects of JAa and AFt–JAa on the cell cycle (a) and apoptosis (b) of SKBR-3 and MDA-MB-231 cells. Cells were treated with JAa or AFt–JAa (0.2 μM, 0.4 μM JAa), AFt (0.0033 μM) for 48 h. Significant differences are expressed as *p < 0.05, **p < 0.01, ***p < 0.001, and ****p < 0.0001.

To ensure that the mechanism of action of JAa is preserved following its encapsulation within AFt, cell cycle perturbation and apoptosis-induction were examined in TfR1+ SKBR-3 and MDA-MB-231 following exposure to JAa or AFt–JAa for 48 h. Consistent with a mechanism of action involving microtubule disruption^27,28^ and corroborating previous reports, 0.4 μM JAa evoked SKBR-3 G2/M cell cycle accumulation (∼26%). AFt–JAa-treatment led to ∼13% increase of G2/M events compared to the naked agent at 0.2 μM (Figure 5a). A corresponding depletion of events in the G1 phase was observed: ∼23% for naked JAa (0.2 μM) and ∼45% for AFt–JAa (at 0.2 μM JAa). This can be explained by apoptosis, a consequence of DNA caspase cleavage in G2/M-blocked cells.

Indeed, significant sub-G1 events were recorded following treatment of SKBR3 cells with all treatment concentrations used in our study. AFt-encapsulation led to a significant increase of sub-G1 cell cycle phase in SKBR-3 cells: by 145% for naked JAa and 157% for AFt–JAa 0.2 μM JAa). Naked JAa (0.4 μM JAa) and AFt–JAa (0.4 μM JAa) caused comparable changes to G1 and sub-G1 events (Figure 5); in MDA-MB-231 cells, exhibiting lower sensitivity to JAa, with enhanced cell cycle perturbation following treatment with AFt–JAa where significant change of the G1 events was observed (*p* < 0.05 for 0.2 μM JAa). An apoptotic mode of cell death was confirmed by annexin-V assays (Figures 5, Supporting Information, S14 and S15).

**Figure 6:**
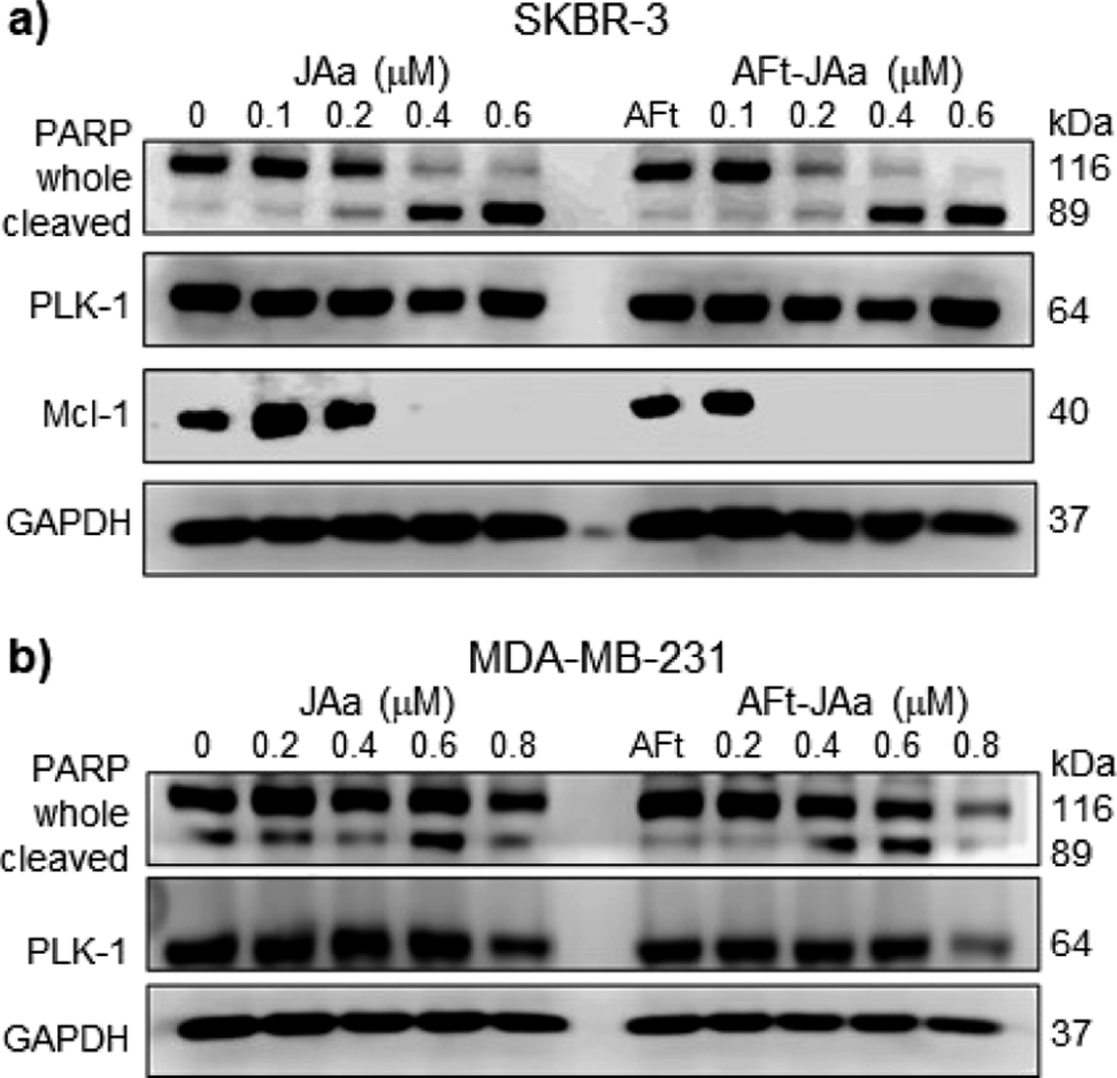
Protein expression in (a) SKBR-3 and (b) MDA-MB-231 cell lysates following 72 h of exposure of cells to JAa and AFt–JAa at different concentrations, AFt (0.0068 μM) and media only. Dose-dependent increase in cleaved PARP and obvious downregulation of whole PARP in both cell lines is observed. Downregulation of survival protein Mcl-1 is evident in SKBR-3 lysates following exposure of cells to JAa (≥0.4 μM) and AFt–JAa (≥0.2 μM). Downregulation of oncogenic PLK-1 was detected in MDA-MB-231 lysates following exposure of cells to naked or AFt-encapsulated JAa (0.8 μM). SI7 demonstrates the densitometry of target proteins’ band intensities expressed as a ratio of target protein to loading control (GAPDH) band intensity using FIJI software.

These results suggest enhanced cell cycle perturbation and apoptosis-induction following AFt-encapsulation of JAa. Apoptosis-induction in breast cancer cells was validated by western blot evaluation of proteins associated with apoptosis or survival. Following 72 h of exposure of SKBR-3 and MDA-MB-231 to Jaa or Aft–Jaa (0.1–0.8 μM Jaa), PARP and Mcl-1 expression was investigated (Figure 6). In addition, PLK-1 expression was interrogated. In SKBR3 lysates, loss of whole PARP, dose-dependent PARP cleavage, and down-regulation of Mcl-1 were consistently observed with AFt–JAa treatment ≥0.2 μM (*p* < 0.0001) and naked JAa ≥ 0.4 μM (Figures 6a and Supporting Information, S16).

Following 48 h of exposure, a significant increase (*p* < 0.001) in early apoptotic populations was observed in SKBR-3 cells (18% for JAa and 21% for AFt–JAa at 0.2 μM JAa). A similar trend was observed for MDA-MB-231 cells with 0.4 μM JAa treatment increasing late apoptosis populations (*p* < 0.05). In less sensitive MDA-MB-231 cell lysates, enhanced downregulation of whole PARP and PLK-1 was detected at 0.8 μM JAa following its AFt-encapsulation (*p* < 0.001; Figures 6b and Supporting Information, S17). The role of PARP enzymes in regulating cellular proliferation, survival, and death is well recognized,^44^ and proteolytic cleavage of PARP by caspases is considered a hallmark of apoptosis.^45^ Downregulation of survival onco-protein Mcl-1 is known to induce apoptosis in cancer cells.^46,47^ PLK1, an important regulator in cancer initiation and progression, is highly expressed in certain tumors, and its inhibition is thought to trigger apoptosis.^48,49^ PLK-1 oncoprotein is a validated cancer target^49^ and molecular target of JA and JB.^27,28^ Taken together, PARP cleavage in both cell lines, loss of Mcl-1 expression in SKBR-3, and inhibition of PLK1 in MDA-MB-231 suggest apoptosis-induction through different pathways in these cell lines and support the results of Annexin V assays. The mechanisms of activity and cell death observed following treatment with the AFt-encapsulated agent are consistent with that reported for naked jerantinines;^27,28^ however, activity is significantly improved as a consequence of selective and enhanced uptake of AFt-encapsulated JAa in TfR1-overexpressing breast cancer cell lines. Our preliminary *in vivo* results confirm biocompatibility of AFt carrier and tumor growth delay following treatment with an Aft-encapsulated drug and merit further detailed evaluation.

## Conclusions

In this study, we describe successful encapsulation of JAa within the core of AFt optimizing the reassembly route. Encapsulation evaluation revealed ∼70% AFt protein recovery and entrapment of ∼120 JAa molecules. The final formulation significantly enhanced the activity of JAa and selectivity in TfR1+ breast cancer cell lines irrespective of their phenotype. The potentiation of Jaa’s activity, demonstrated by potent growth inhibitory, clonogenic and apoptosis assays, was related to increased (and selective) recognition and uptake of AFt–JAa by TfR1, leading to enhanced accumulation of JAa within cancer cells without altering its mechanism of action. AFt alone had negligible effects on cells, indicating a biocompatible delivery system. By exploiting the intrinsic binding properties of AFt to TfR-1 and the breast cancer cells’ enhanced TfR-1 expression, AFt-encapsulation of JAa confers a significant degree of cancer-selectivity. Corroborating this thesis, we recently demonstrated that TfR1-recognition can be abolished through mutagenesis of AFt at the TfR1 binding recognition site (L. Ferreira, manuscript in preparation). However, TfR-1 expression may be upregulated in benign proliferative disease as shown in MCF-10A cells; therefore, further refinement for tumor-targeting is necessary. Development of affibody-conjugated AFt delivery capsules that target proteins overexpressed in certain mammary tumors remains a goal of current research, ultimately to enhance survival rates and improve prognoses in breast cancer patients. In this work, we report a robust AFt-reassembly route to JAa-encapsulation and present evidence that AFt–JAa provides a potential treatment for intractable, drug-resistant mammary carcinomas, worthy of further preclinical development.

## Supporting Information Available

Information about measuring the concentration of the protein and jerantinine A acetate; assessment of protein recovery and EE %; growth-inhibitory assessment of AFt–JAa; release study of jerantinine A acetate from AFt; stability study; cell culture studies; and densitometry and semi-quantitative analyses (PDF)
