# Correction to “Apoferritin-Encapsulated Jerantinine A for Transferrin Receptor Targeting and Enhanced Selectivity in Breast Cancer Therapy”

**DOI:** 10.1021/acsomega.3c09291

**Published:** 2023-12-20

**Authors:** Haneen Abuzaid, Salah Abdelrazig, Lenny Ferreira, Hilary M. Collins, Dong-Hyun Kim, Kuan-Hon Lim, Toh-Seok Kam, Neil R. Thomas, Lyudmila Turyanska, Tracey D. Bradshaw

**Affiliations:** † School of Pharmacy, Biodiscovery Institute, The University of Nottingham, University Park, Nottingham NG7 2RD, U.K.; †† School of Chemistry, Biodiscovery Institute, The University of Nottingham, University Park, Nottingham NG7 2RD, U.K.; ‡ The University of Nottingham Malaysia, Block B, Malaysia Campus, Jalan Broga, 43500 Semenyih, Selangor, Malaysia; § Department of Chemistry, Faculty of Science, The University of Malaya, 50603 Kuala Lumpur, Malaysia; ∥ Faculty of Engineering, The University of Nottingham, Additive Manufacturing Building, Jubilee Campus, University Park, Nottingham NG7 2RD, U.K.

Neil R. Thomas was added as an author. The change in authorship is reflected in the authorship of this Correction.

