# Supplementary material for: Apoferritin-Encapsulated Jerantinine A for Transferrin Receptor Targeting and Enhanced Selectivity in Breast Cancer Therapy
Source: ACS Omega. 2022 Jun 13;7(25):21473–82. doi: 10.1021/acsomega.2c00997 (PMC9244903; doi:10.1021/acsomega.2c00997)
Supplement: Supplementary file 1 — ao2c00997_si_001.pdf [file ao2c00997_si_001.pdf]

SUPPORTING INFORMATION

# Apoferritin-encapsulated Jerantinine A for transferrin receptor-targeting and enhanced selectivity in breast cancer therapy

Haneen Abuzaid[1], Salah Abdelrazig[1], Lenny Ferreira[1], Hilary M. Collins[1], Dong-Hyun Kim[1], Kuan-Hon Lim[2], Toh-Seok Kam[3], Lyudmila Turyanska[4] and Tracey D. Bradshaw[1]

[1]School of Pharmacy, University of Nottingham, Nottingham, NG72RD, UK; [2]School of Pharmacy, University of Nottingham Malaysia, Jalan Broga, 43500 Semenyih, Selangor, Malaysia; [3]Department of Chemistry, Faculty of Science, University of Malaya, 50603 Kuala Lumpur, Malaysia; [4]Faculty of Engineering, University of Nottingham, Nottingham, NG72RD, UK.

Correspondence: Tracey.Bradshaw@nottingham.ac.uk

Keywords: jerantinine, drug delivery, apoferritin, transferrin receptor, breast cancer

## SI1. Measuring concentration of protein and jerantinine A acetate (JAa)

The concentrations of all proteins used in this study were calculated using a calibration curve obtained by Bradford analysis (Figure S1a). The concentration of encapsulated JAa was assessed using UV-Vis spectroscopy of JAa solution (Figure S1b) with different concentrations. Based on these results, the calibration curve (Figure S1c) was generated and used to calculate the concentration of encapsulated JAa.

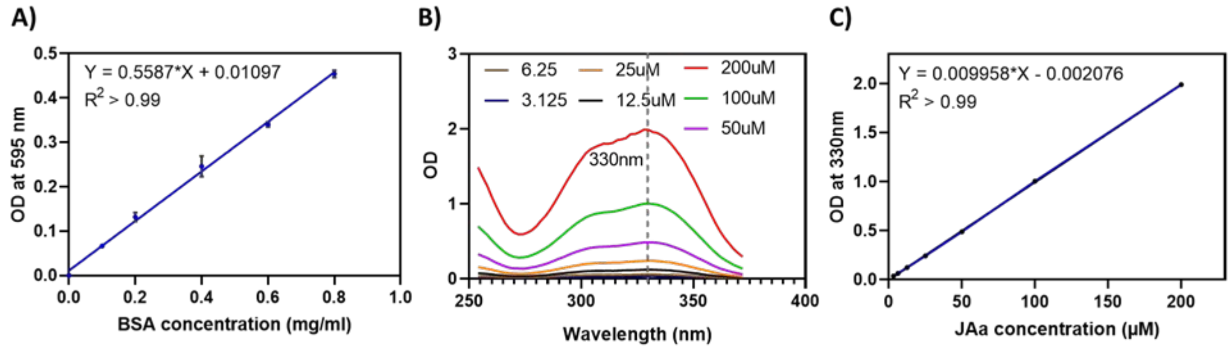


**Figure S1. a)** The standard curve of BSA, R2 > 0.99. **b)** Absorbance spectra of JAa with different concentrations, with absorbance peak contred at 330 nm. **c)** Calibration curve showing integrated intensity of JAa absorbance peak at 330 nm, R2 > 0.99.

## SI2. Assessment of protein recovery and encapsulation efficiency EE%

To optimize the encapsulation methods, the percentage of protein recovery was calculated using Equation (1):

$$concentrate\ recovery\ \% = 100 * \frac{Wc * Cc}{W0 * C0}, \tag{S1}$$

where $W_c$ is the weight of the formulation after purification, $C_c$ is the concentration of the formulation after purification, $W_0$ is the weight of formulation before purification and $C_0$ is the concentration of the formulation before purification. Encapsulation efficiency (EE%) is defined as the ratio between the encapsulated and the original amount of the drug added to the formulation:

$$EE\% = \frac{amount\ of\ encapsulated\ drug}{amount\ of\ added\ drug} 100\% \tag{S2}$$

## SI3. Growth-inhibitory assessment of AFt-JAa

Table S1 summarizes the information about the cell lines used.

**Table S1**. Cell lines used and their supplemented media

| Cell line | Medium used | Supplements |
|---|---|---|
| MCF-7 | RPMI-1640 | 10% FBS |
| BT-474 | DMEM-high glucose | 10% FBS |
| MDA-MB-468 | MEM | 10% FBS |
| SKBR-3 | McCoy's 5a | 10% FBS |
| MDA-MB-231 | MEM | 10% FBS |
| MCF-10A | DMEM/F12 | 2.5% Horse serum, 20 ng/ml EGF, 0.5 mg/ml hydrocortisone, 10 µg/ml insulin, 1% penicillin-streptomycin |
| MRC-5 | MEM | 10% FBS, 1% 1M HEPES, 1% 200 mM L-glutamine, 1% 0.1 mM NEA, 7.5% sodium bicarbonate,1% penicillin-streptomycin |

The test agent concentration required to achieve 50% growth inhibition, $GI_{50}$ were calculated using Equation (S3):

$$GI_{50} = \left(\frac{A_H - AGI_{50}}{A_H - A_L} * (C_H - C_L)\right) + C_L \,, \tag{S3}$$

where $AGI_{50} = \frac{A_1 - A_0}{2} + A_0$ , $A_1$ is absorbance in control (untreated) wells; $A_0$ is absorbance at T0, $A_H$ and $A_L$ are absorbances higher and lower than $AGI_{50}$, at concentrations $C_H$ and $C_L$, respectively. In clonogenic assays, plating efficiency (PE%) and survival fractions (SF%) of colonies were calculated using Equations (S4) and (S5), respectively:

$$PE\% = \frac{Number\ of\ colonies\ counted}{Number\ of\ cells\ plated} 100\% \tag{S4}$$

$$SF\% = \frac{PE\ of\ treated\ sample}{PE\ of\ control} 100\% \tag{S5}$$

## SI4. Release study of jerantinine A acetate (JAa) from apoferritin (AFt)

Release studies were performed using Slide A-Lyzer MINI Dialysis Device (Thermo Scientific, PC-8840, 0.5 mL) with 10 K MWCO); 100 mM sodium acetate buffer (NaOAc) adjusted to pH 5.3 by acetic acid; phosphate-buffered saline (PBS, pH 7.4). The formulation (*n* = 4) was placed into the dialysis device and kept at 37 °C either in NaOAc or PBS. Direct and indirect samples were collected at different time intervals of 0 h, 1 h, 3 h, 6 h, 24 h (Figure S2). JAa concentration was estimated using the calibration curve obtained after the liquid chromatography-high resolution mass spectrometry (LC-HRMS) analysis. *Indirect samples:* The JAa concentration was estimated after measuring JAa concentrations remaining in the dialysis device. First, samples were treated with 1:5 formic acid: methanol to precipitate and open the AFt cage. Samples were then centrifuged (13,000 rpm; 10 min) and the supernatant was measured using EQMS

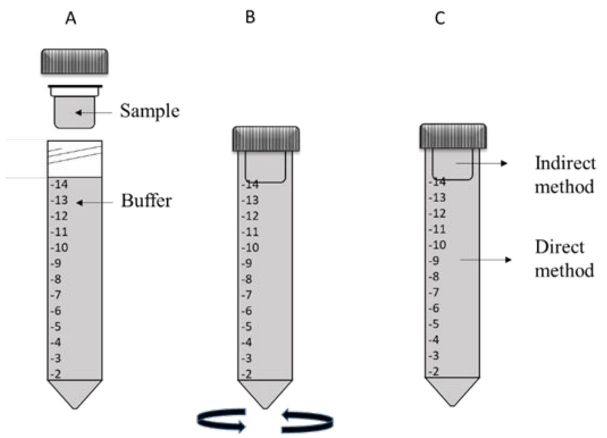


**Figure S2.** Schetch of the release study: (A) Samples dialyzed at pH 5.3 (NaOAc) or PH 7 (PBS) then (B) shaken gently (*T* = 37 °C, 24 h) before (C) direct and inderect samples are collected at specific times (1 h, 3 h, 6 h, and 24 h) for LC-HRMS analysis.

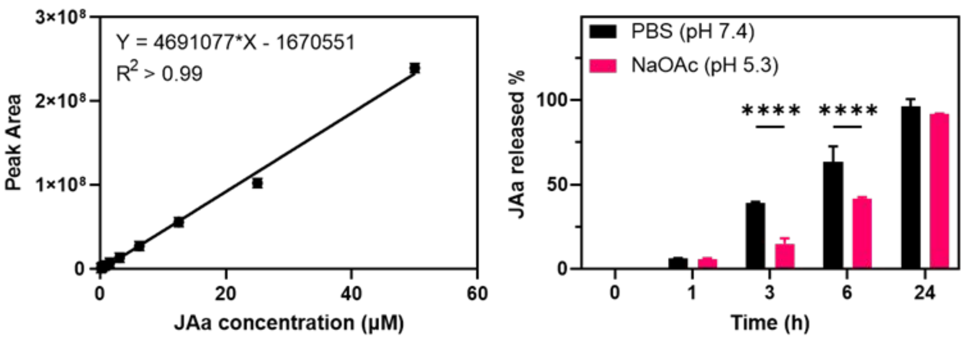


.

**Figure S3.** The standard curve of JAa obtained from LCMS and release profile of JAa from AFt (24 h, $T = 37$ °C) at pH 5.3 and pH 7.4 in four independent trials, points represent means ± SD.

## SI5. Stability study

Stability of the encapsulated agent was assessed using DLS measurements and by monitoring the concentration of the agent (Figure S4).

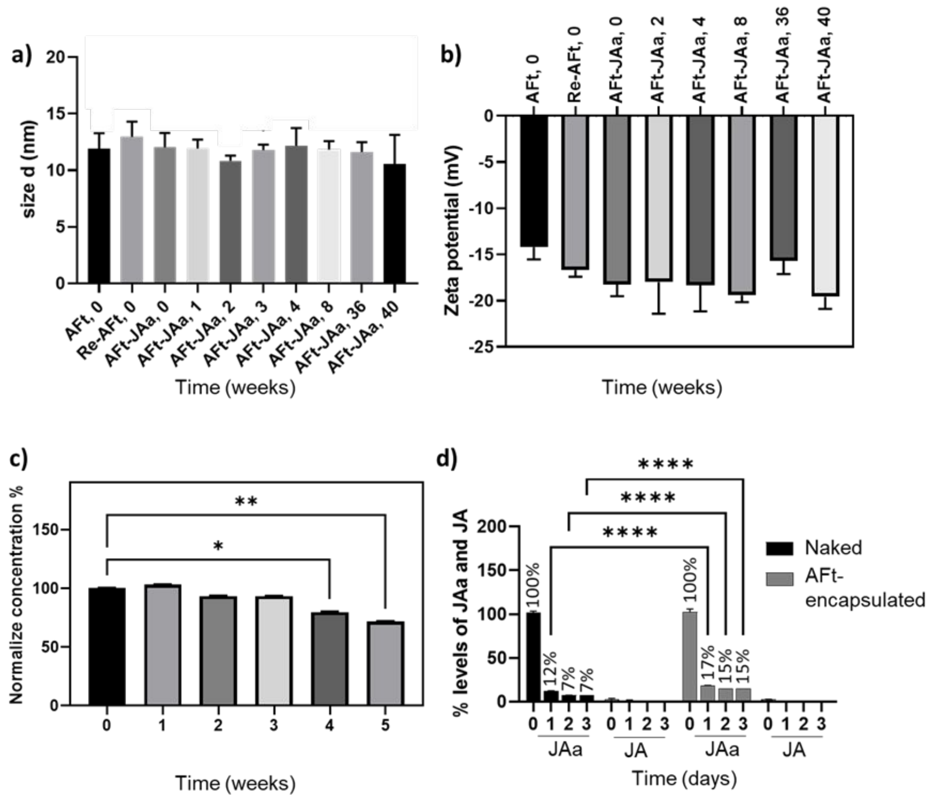


**Figure S4.** Stability study of AFt-encapsulated JAa monitored at storage conditions ($T$ = 4 °C; pH 7.4). Hydrodynamic size (a) and zeta-potential (b) were measured using Malvern zeta sizer Nano ZS. Measurements of AFt-JAa and empty reassembled AFt (Re-AFt) were statistically comparable to AFt stock over 10 months of storage. The stability of encapsulated JAa was assessed by measuring its concentrations by UV-Vis spectroscopy at 330 nm over 5 weeks (c). Stability study of free and AFt-encapsulating JAa (0.4 µM) monitored at treatment conditions ($T$ = 37 °C; over 72 h) in different media using HR-LCMS. Measurements show better stability of JAa following the encapsulation with AFt in 7 independent trials (d).

## SI6. Cell culture studies

Activity and selectivity of the tested agents were assessed using MTT assays (Figures S5-S7), cell count assays (Figure S8), clonogenic (Figure S9), cell cycle analyses (Figures S12-13) and Annexin apoptosis studies (Figures 14-15). The representative MTT growth-inhibitory profiles demonstrate the ability of JAa to inhibit the growth of breast carcinoma cell lines, normal breast cells (MCF-10A) and MRC5 fibroblasts. Cancer-cell selectivity is significantly enhanced after AFt encapsulation of JAa, where significantly smaller numbers of viable cells were counted; perturbation of cell cycles was observed (with SKBR3 G2/M arrest evident; Figure S12) consistent with microtubule disrution, and large cancer cell populations undergoing apoptosis. Enhanced anticancer activity and selectivity with AFt-JAa is attributed to high expression of TfR1 (Figure S10) facilitating uptake of H-AFt (Figure S11) and enapsulated JAa in breast carcinomas.

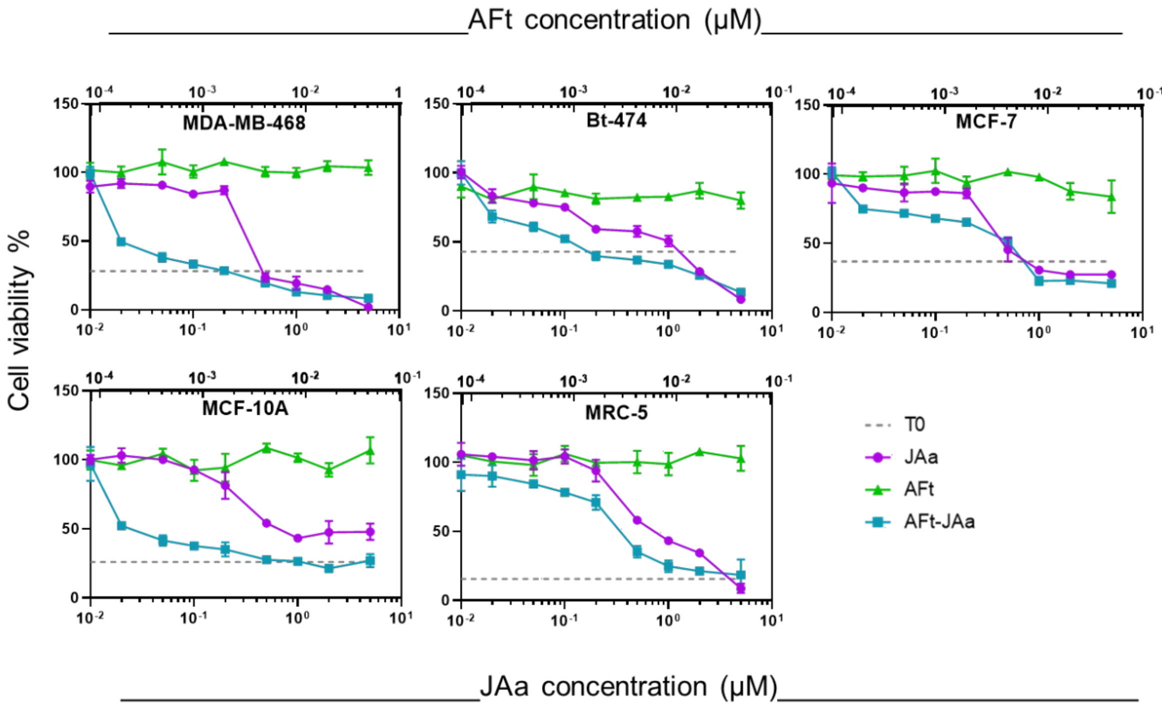


**Figure S5:** Representative MTT graphs from a single trial (n=4 internal replicates) displaying the growth inhibitory properties of apoferritin-encapsulated JAa (AFt-JAa), JAa, and AFt in MDA-468, BT-474, MCF-7, MCF-10A and MRC-5.

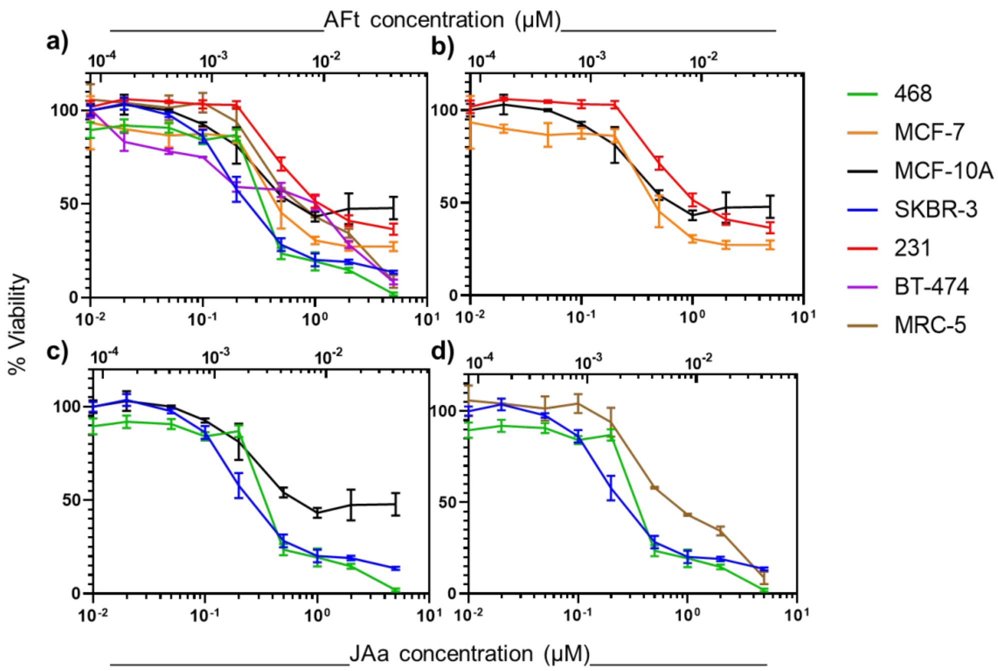


**Figure S6.** Representative MTT growth-inhibitory profiles after treatment with JAa (a). Observed selectivity of JAa at a concentration of 5 µM to inhibit MCF-7 by ~72% and MDA-MB-231 by ~63% compared to inhibit non-cancerous (MCF-10A) µM by ~52% (b). Inhibition of the growth of MDA-MB-468 by 98% and SKBR-3 by ~86%, over non-cancerous MCF-10A (c). MDA-MB-468 by 98% and SKBR-3 show indistinguishable growth inhibition of JAa compared to MRC-5 (d). Cells were seeded in 96-well plates at a density of 3-4 x $10^3$ cells/well, left to adhere for 24 h, treated and incubated for 48 h, except for BT-474 which was treated for 72 h and excluded from this comparison.

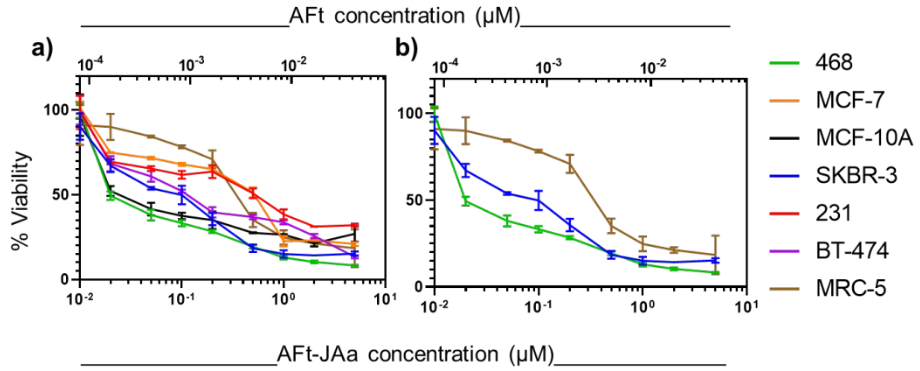


**Figure S7.** Representative MTT growth-inhibitory profiles in all studied cell lines after treating with AFt-JAa (a). Growth inhibition profiles of two TFR+ cells (MDA-MB-231 and SKBR-3) and non-cancerous TFR- MRC-5 cells (b). Cells were seeded in 96-well plates at a density of 3-4 x $10^3$ cells/well. After allowing to adhere (24 h), all cell lines were treated and incubated for 48 h, except for BT-474 which was treated for 72 h.

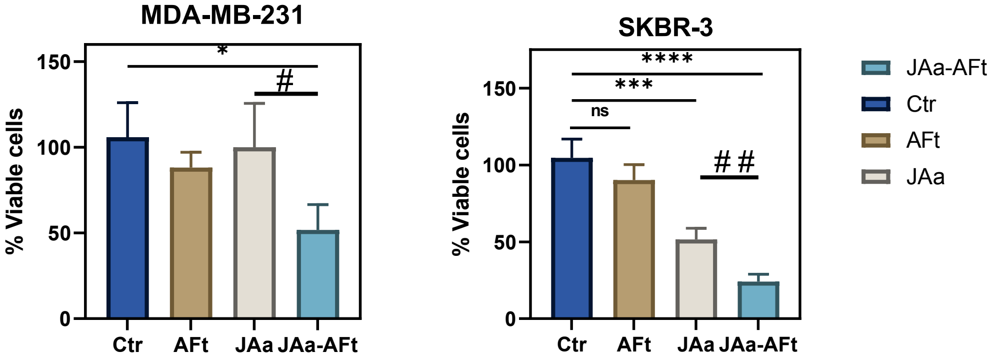


**Figure S8.** *In vitro* cell viability assays in MDA-MB-231 and SKBR-3 following treatment with JAa, AFt-JAa ( 0.2 µM JAa), AFt ( 0.0017 µM) or media alone for 48 h. Cells (2 x $10^4$ cells/well) were seeded in 6-well plates and incubated overnight before the treatment. Cells were harvested stained with trypan blue and counted by haemocytometer.

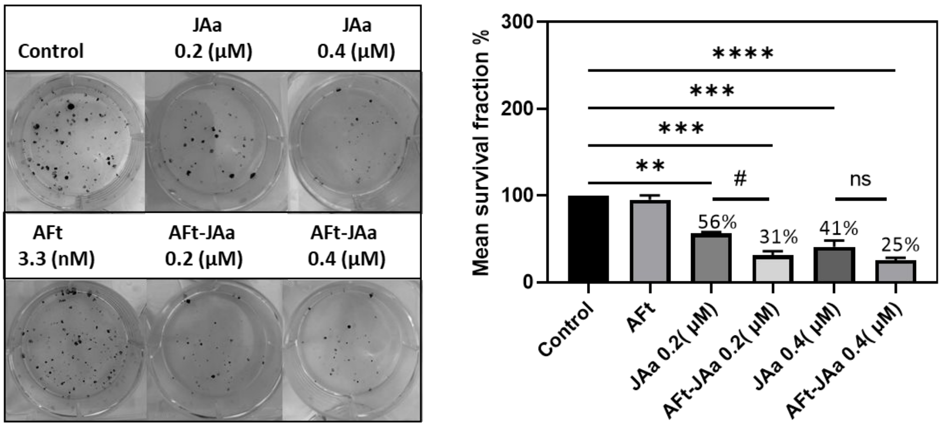


**Figure S9.** Representative BT-474 colonies demonstrating the effect of naked or AFt-encapsulated JAa (0.2 µM and 0.4 µM JAa), AFt (0.0033 µM) or medium alone on BT-474 clonal survival following 48 h exposure. Clonogenic survival fractions are reported as mean ± SD (n=3 internal repeats) and repeated 3 times. Significant differences from the control are expressed as **P < 0.01, ***P < 0.001 and ****P < 0.0001. Significant differences from naked JAa are expressed as #P < 0.05.

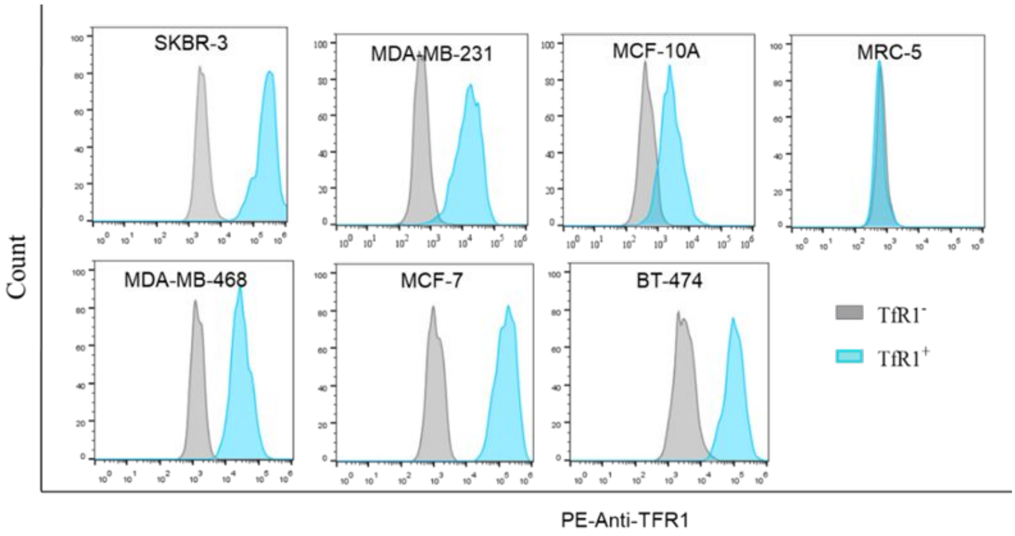


**Figure S10.** Expression of TfR1. Representative flow cytometry plots showing the shift in TfR1 after binding of PE-Anti-TfR1 in breast cancer cell lines in blue (SKBR-3, MDA-231, MCF-7, MDA-MB-468, MCF-7 and BT-474). And a non-detectable shift in MRC-5 cells.

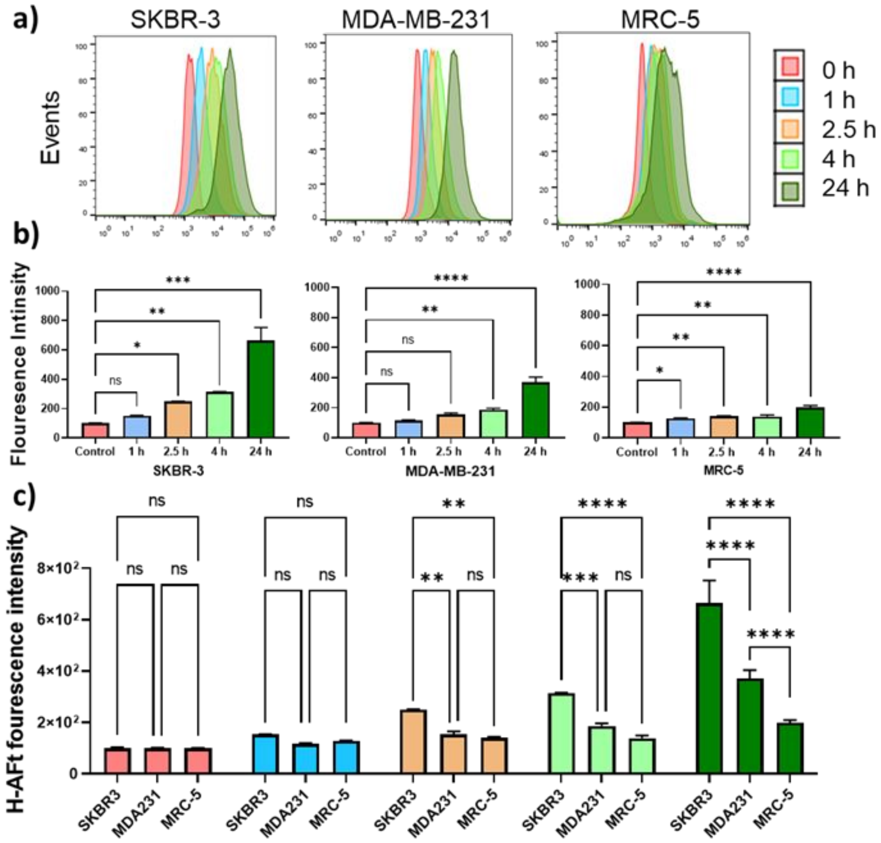


**Figure S11.** Cellular uptake study of SKBR-3, MDA-231 and MCF-7 to 5-carboxyfluorescein-conjugated-human-apoferritin ( 40 nM) following 1 h, 2:30 h, 4 h and 24 h of exposure. Representative flow cytometry plots showing the shift in H-AFt fluorescence intensity indicating its cellular uptake (a). statistical analyses of H-AFt uptake with time compared to control at (0 time) of exposure for each cell line (b). statistical analyses of H-AFt uptake with time of TfR1+ cells (SKBR-3 and MDA-MB-231) compared to TfR1- cells (non-cancerous MRC-5) at all exposure time (c). Data are reported as median ± SD (n

= 3) and repeated 3 times. Significant differences from the control are expressed as *P < 0.05, **P < 0.01, ***P < 0.001 and ****P < 0.0001.

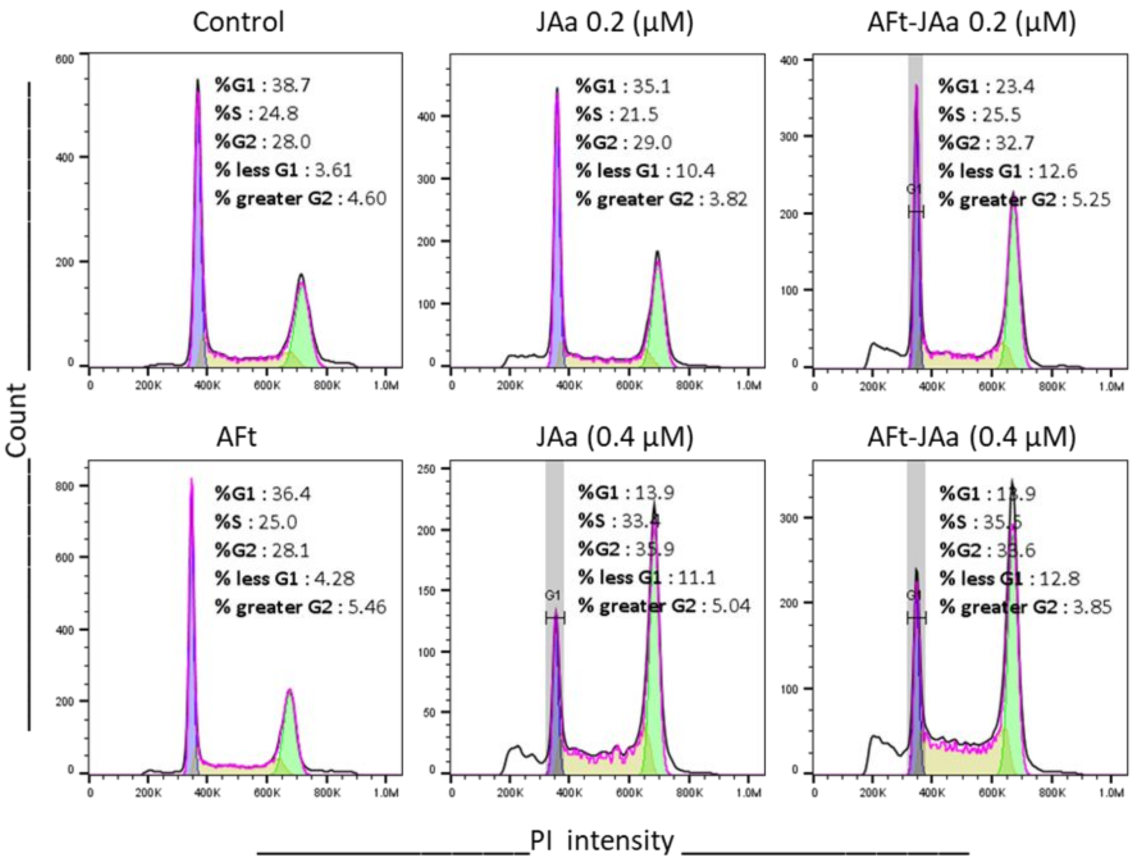


**Figure S12.** Representative cell cycle histograms from a single trial of SKBR-3 cells following treatment with JAa, AFt-JAa ( 0.2 µM JAa) and ( 0.4 µM JAa), AFt ( 0.0033 µM) or media alone for 48 h. Cells (1 x $10^5$ cells/well) were seeded in 6-well plates and incubated overnight before the treatment.

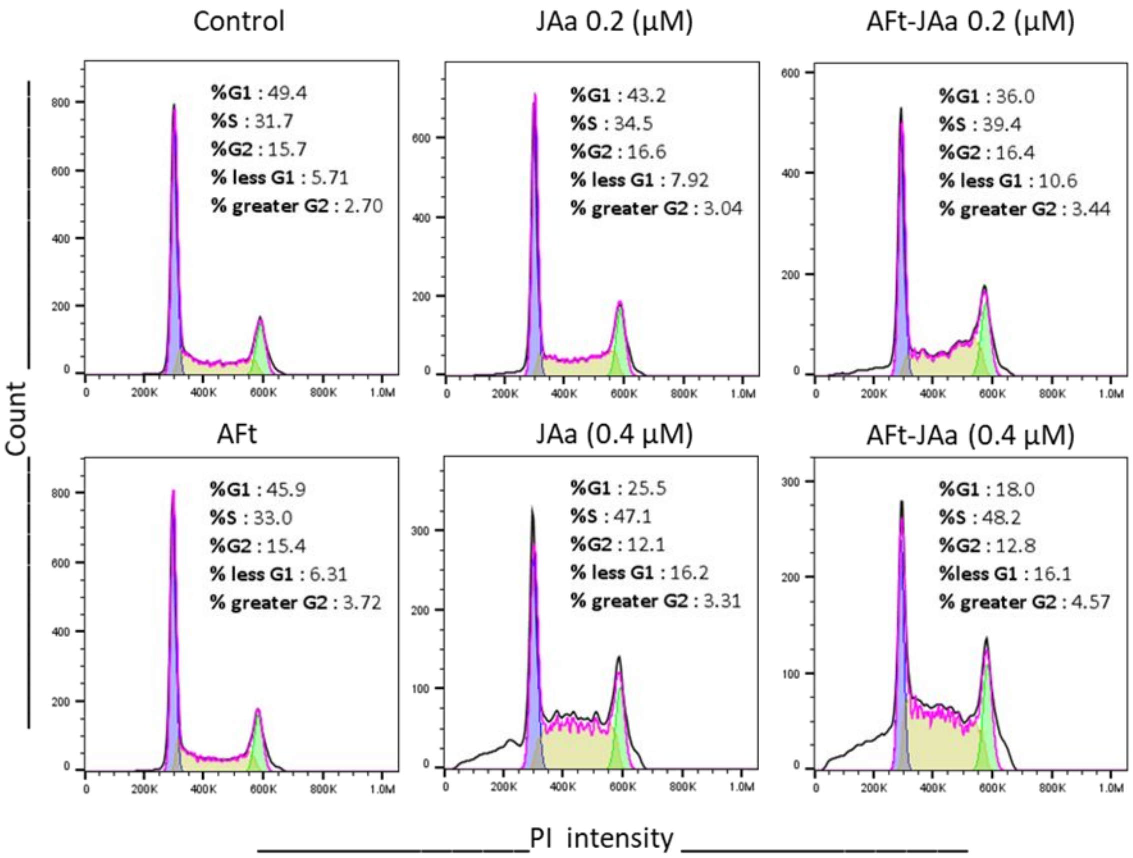


**Figure S13.** Representative cell cycle histograms from a single trial of MDA-MB-231 cells following treatment with JAa, AFt-JAa ( 0.2 µM JAa) and ( 0.4 µM JAa), AFt ( 0.0033 µM) or media alone for 48 h. Cells (1 x $10^5$ cells/well) were seeded in 6-well plates and incubated overnight before the treatment.

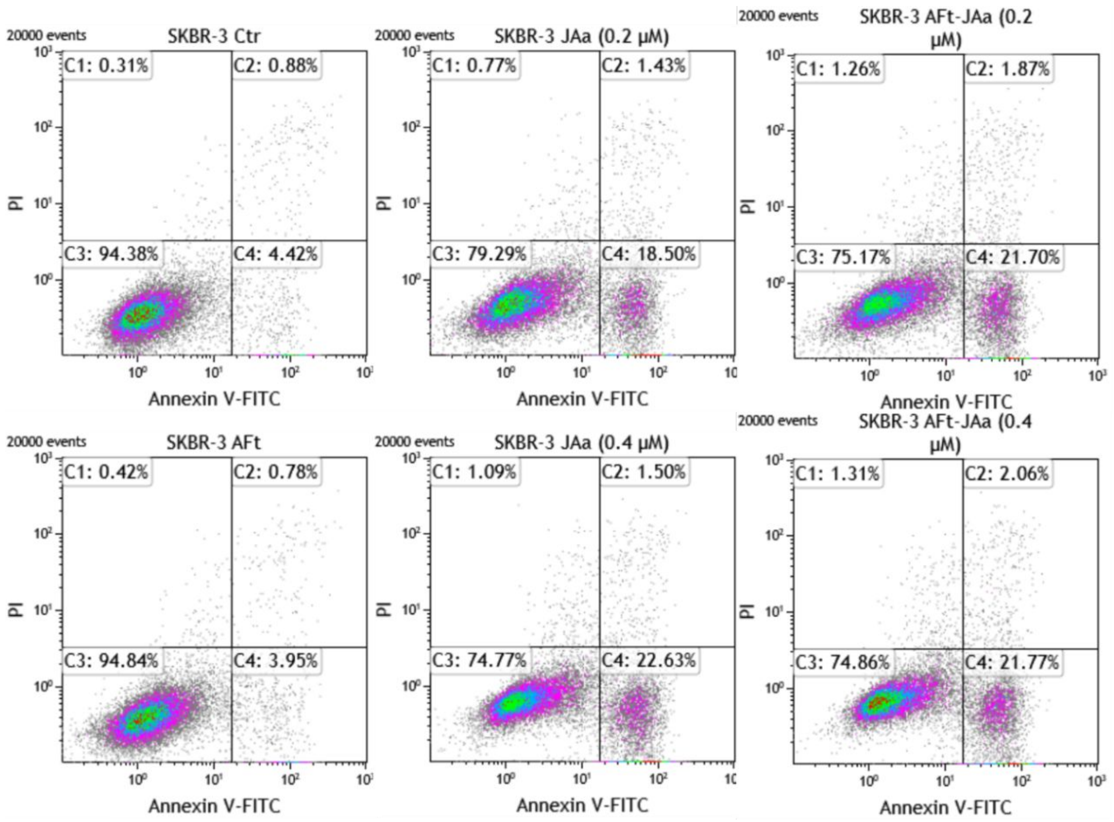


**Figure S14.** Representative apoptosis quadrant plots from a single trial of SKBR-3 cells illustrating apoptotic effects following treatment with JAa, AFt-JAa ( 0.2 µM JAa) and ( 0.4 µM JAa), AFt ( 0.0033 µM) or media alone for 48 h. Cells (1 x $10^5$ cells/well) were seeded in 6-well plates and incubated overnight before the treatment.

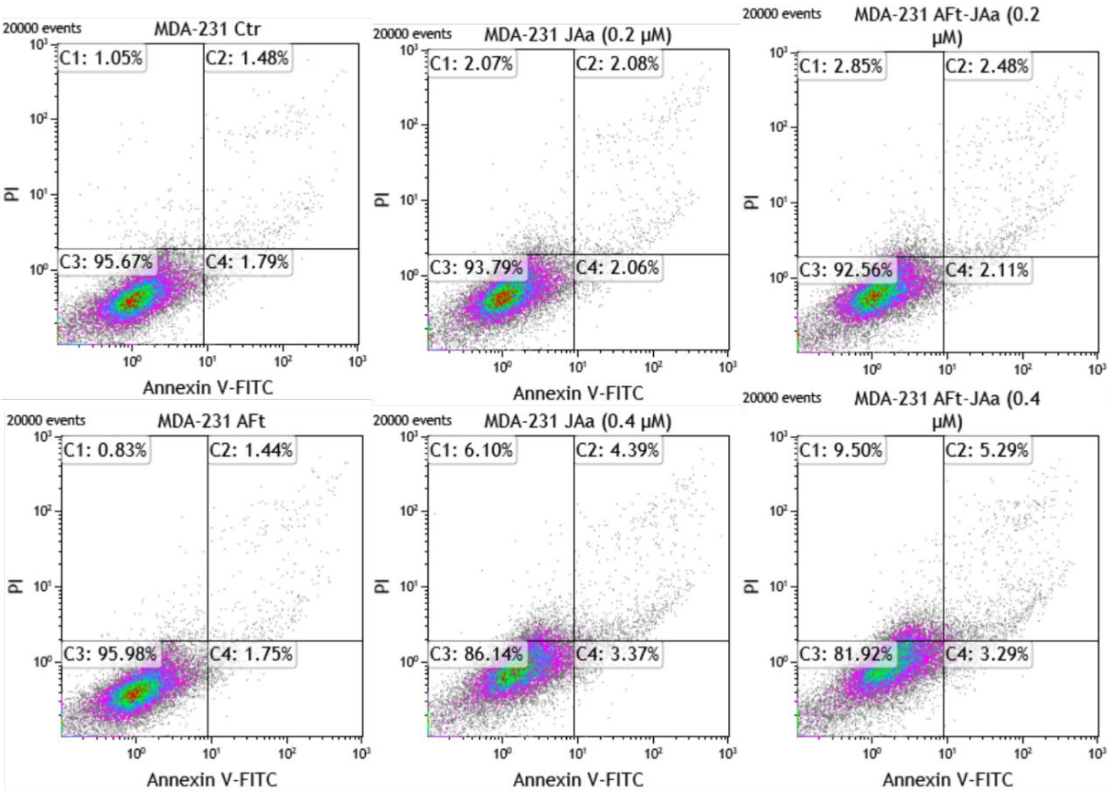


**Figure S15.** Representative apoptosis quadrant plots from a single trial of MDA-MB-231 cells illustrating apoptotic effects following treatment with JAa, AFt-JAa ( 0.2 μM JAa) and ( 0.4 μM JAa), AFt ( 0.0033 μM) or media alone for 48 h. Cells (1 x $10^5$ cells/well) were seeded in 6-well plates and incubated overnight before the treatment.

## SI7. Densitometry and semi-quantitative analyses

The apoptosis induced by free- and AF-encapsulated JAa was corroborated by Western blot studies. The densitometry semi-quantitative analyses for SKBR-3 (Figure S16) lysates revealed a dose-dependent cleavage of PARP and inhibition of anti-apoptotic MCl-1 expression in SKBR-3 cells, and a down-regulation of PLK-1 oncogenic kinase with the highest-used JAa concentration in MDA-MB-231 (Figure S17).

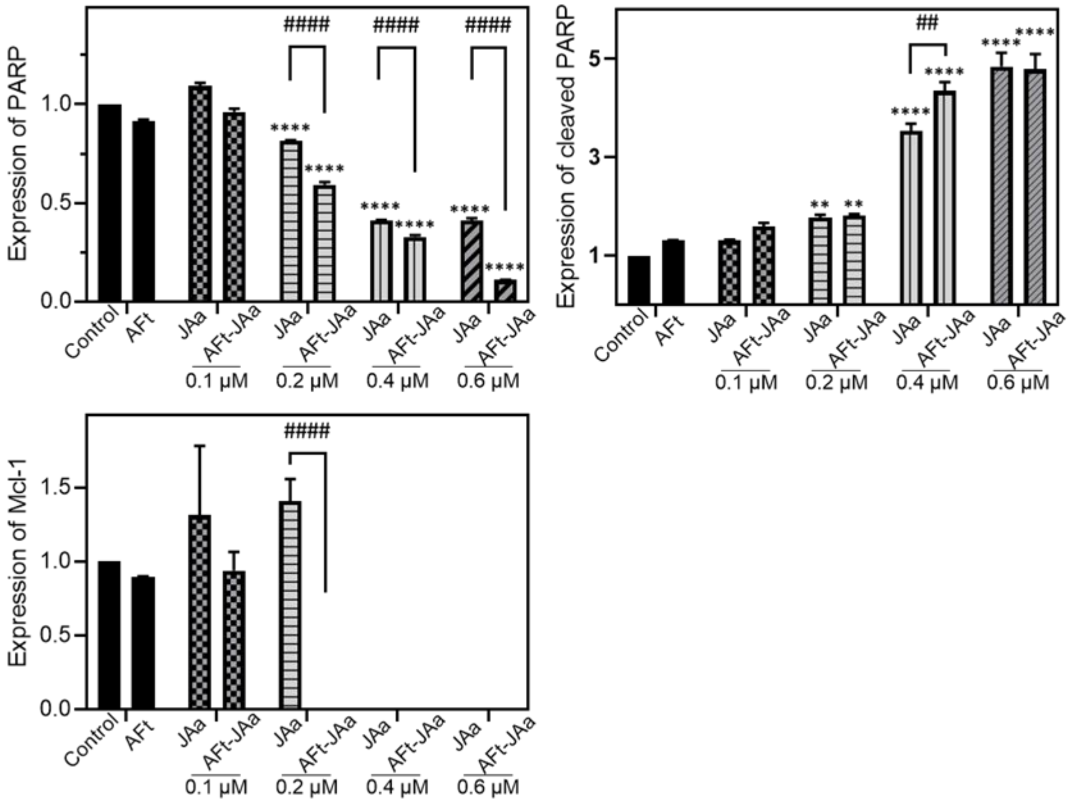


**Figure S16.** Densitometry semiquantitative analysis of SKBR-3 cells.

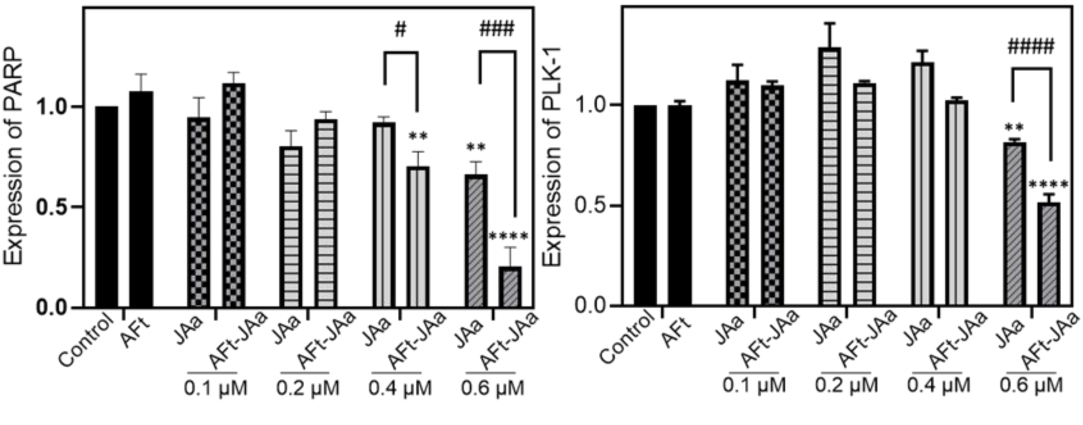


**Figure S17**. Densitometry semiquantitative analysis of MDA-MB-231 cells. Significant differences are expressed as $*p < 0.05$, $**p < 0.01$, $***p < 0.001$, $****p < 0.0001$; $^{\#}p < 0.05$, $^{\#\#}p < 0.01$, $^{\#\#\#}p < 0.001$, $^{\#\#\#\#}p < 0.0001$.
